# The potential of new nicotine and tobacco products as tools for people who smoke to quit combustible cigarettes – a systematic review of common practices and guidance towards a robust study protocol to measure cessation efficacy

**DOI:** 10.1186/s12954-024-01047-1

**Published:** 2024-07-05

**Authors:** Nikola Pluym, Therese Burkhardt, Gerhard Scherer, Max Scherer

**Affiliations:** ABF Analytisch-Biologisches Forschungslabor GmbH, Semmelweisstr. 5, 82152 Planegg, Germany

**Keywords:** Smoking cessation efficacy, Smoking abstinence, Electronic cigarettes, Heated tobacco products, Nicotine pouches, Compliance monitoring

## Abstract

New types of nicotine and tobacco products like electronic cigarettes (ECs), heated tobacco products or nicotine pouches have been discussed as less harmful alternatives to combustible cigarettes and other toxic forms of tobacco products. Their harm reduction potential lay in the efficient transition away from smoking to those new products. Numerous studies addressing the cessation efficacy of ECs have been published with contradictory outcomes. Yet, a comprehensive Cochrane review concluded with high certainty on the cessation efficacy of ECs. This prompted us to perform a review to identify weaknesses in common study designs and to summarize best practices for the study design on the potential of new nicotine products as cessation aids. 120 articles retrieved from Medline were found to be eligible. Most of the studies in the field were interventional trials while observational studies played a minor role in the evaluation of smoking cessation. Efficacy was predominantly assessed for ECs in 77% of the reports while heated tobacco (17%) and non-combustible products (11%) were less frequently investigated up to now. Measures to determine the efficacy were questionnaire-based assessments as well as use documentation/prevalence and abstinence rates. Studies varied largely in their duration and sample size with medians of 3 months and 156.5 participants, respectively.

With the help of this review, we identified several weaknesses in the common study designs. One major limitation in longitudinal trials was the lack of compliance measures suited to verify the use status over longer time periods, relying solely on self-reports. Moreover, the motivation of the participants to quit was rarely defined and a profound familiarization period was not taken into account for the majority of the studies. To what extent such weaknesses influence the outcome of the studies was beyond the scope of this review. We encourage researchers to consider the recommendations which resulted from this review in order to determine the abuse liability and cessation efficacy of the products in a more robust manner. Finally, we like to call attention to the missing data for low- and middle-income countries which would require quitting strategies most urgently to combat the tobacco smoking epidemic.

**Supplementary Information:**

The online version contains supplementary material available at 10.1186/s12954-024-01047-1.

## Introduction

Smoking combustible cigarettes (CCs) has been and continues to be an enormous public health burden worldwide. In 2019 alone, there were more than 1.1 billion people who smoke and the habit accounted for roughly 7.7 million premature deaths globally [1]. In order to end this tobacco epidemic, a substantial number of current consumers of CCs need to quit. Although the majority of people who smoke are willing to quit, they find it very hard to do so permanently [2]. Quit success rates without support are around 50% at one week and less than 5% after one year [3]. While behavioral support and nicotine replacement therapies (NRTs) like nicotine gums and patches increased the chances of quitting, long-term abstinence remained rather low [4–6]. Clearly, NRTs did not work for everyone and additional cessation aids were needed for addressing the sensory stimulation or haptic properties that provide rewarding effects to the consumers of CCs besides the intake of nicotine [7].

Over the past 20 years, the tobacco landscape has changed drastically with several new emerging product categories available such as e-cigarettes (ECs) or heated tobacco products (HTPs). These combustion-free products bear the potential to reduce the exposure to toxic chemicals present in tobacco smoke while delivering nicotine to the consumer in a similar way to smoking in contrast to NRTs [8, 9]. Swedish snus as a smokeless tobacco product meanwhile almost completely replaced smoking in Sweden, resulting in the lowest tobacco smoking-related mortality in the EU [10, 11].

Altogether these alternatives show a significantly reduced exposure to harmful and potentially harmful constituents of tobacco smoke having the potential to serve as harm reduction tools if a CC consumer is able to switch completely away from smoking [12, 13]. A recent Cochrane review concluded that people who smoke are more likely to quit smoking for at least 6 months using ECs with nicotine compared to NRTs or ECs without nicotine. The meta-analysis showed high-certainty evidence that ECs with nicotine are a more efficacious cessation tool than NRTs [14]. Yet, several studies and previous reviews have reported contradictory findings [15–17]. The question is, why are there such discrepancies in the conclusions when it comes to the use of ECs for quitting? One factor is product evolution resulting in a better bioavailability of nicotine in newer devices as shown by the nicotine pharmacokinetics. Consequently, more recent studies may come to a different outcome than older studies with first generation devices. However, some studies simply may not have been designed appropriately to adequately determine the products’ cessation efficacy. Evidently, poorly designed studies will likely lead to false conclusions. The authors of the Cochrane review included 78 studies over a time period of approximately ten years, comprising about 22,000 participants. Much less data is available for other product categories like HTPs or oral nicotine pouches (ONPs). Recently, Caponnetto et al. published the first study evaluating the efficacy of HTPs for cessation which was comparable to ECs. They concluded that further studies will be needed to substantiate their findings [18].

This prompted us to perform a comprehensive literature review on state-of-the-art clinical study designs addressing product use behavior and cessation efficacy in order to identify the most important characteristics for answering this research question. Moreover, this review shall detect limitations and gaps in common designs, which may undermine the conclusiveness of a study with the ultimate aim to come up with general recommendations for a robust study design. However, the question how these weaknesses impact the validity of the study data has not been evaluated in this review. In order to determine the causality between study design and data accuracy a meta-analysis of the data would be more appropriate which was beyond the scope of our review.

While there may be no protocol or study that will be an all-in-one solution, this review provides recommendations for cessation studies generating robust data with a low risk of bias, so that high certainty evidence for new nicotine/tobacco products can be obtained (hopefully) with a lower number of studies and participants involved.

## Methods

This review was conducted in accordance with PRISMA guidelines [19].

### Search strategy

A Medline database search was conducted to retrieve suitable studies/publications between 2014 and 03-November-2022. For filters and Boolean operators see Supplementary Information. In addition, studies which were included in the Cochrane review on ECs for smoking cessation and met the eligibility criteria were also considered.

### Eligibility criteria

The study characteristics design, location, sample size, duration, endpoints were not considered for eligibility. The following inclusion criteria were applied:

- At least one subgroup in the study had to be users of a new nicotine product (EC, HTP, smokeless tobacco, ONP) or randomized to one of these products in interventional trials.
- Healthy adults (18 + years of age).
- Studies that assessed participants’ behavior (use behavior, withdrawal symptoms), and/or cessation efficacy and/or a risk/exposure assessment by measures listed in Table 1.

Exclusion criteria were:

- Studies in ill cohorts (e.g. COPD, asthma) or focusing on participants with co-use disorders (e.g. alcoholism or other drugs).
- Meta-analyses and review articles.

### Selection process

Title and abstract of the articles retrieved by the initial search were screened with respect to the inclusion and exclusion criteria. Full texts were then acquired for the selected, relevant articles. In the next step of the article inspection, the information suitable for generating this review was extracted from the full text and imported into a general evidence table (see Supplementary Information). This working file formed the basis for the literature-derived information provided in this review.

### Data extraction and analysis

The key information retrieved from the literature was collected in an evidence table structured as follows:

- First author of the study.
- Number of study participants.
- Product use status of the study participants at study start.
- Country in which the study was conducted.
- Brief description of the study design including study type (interventional/observational, cross-sectional, longitudinal, randomized controlled trial, cross-over design).
- Study duration.
- Compliance monitoring by biochemical verification (Y/N); if yes, the biomarker is given.
- Familiarization to the product either by a training session or a set period of use before study start.
- Investigated product.
- Endpoints assessed to determine the cessation efficacy and use behavior.
- Biomarkers of exposure (BoE) and biomarkers of potential harm (BoPH) assessed to investigate exposure and risk.

Data extraction was performed by N.P. and checked by T.B. for completeness.

## Results

**Fig. 1:**
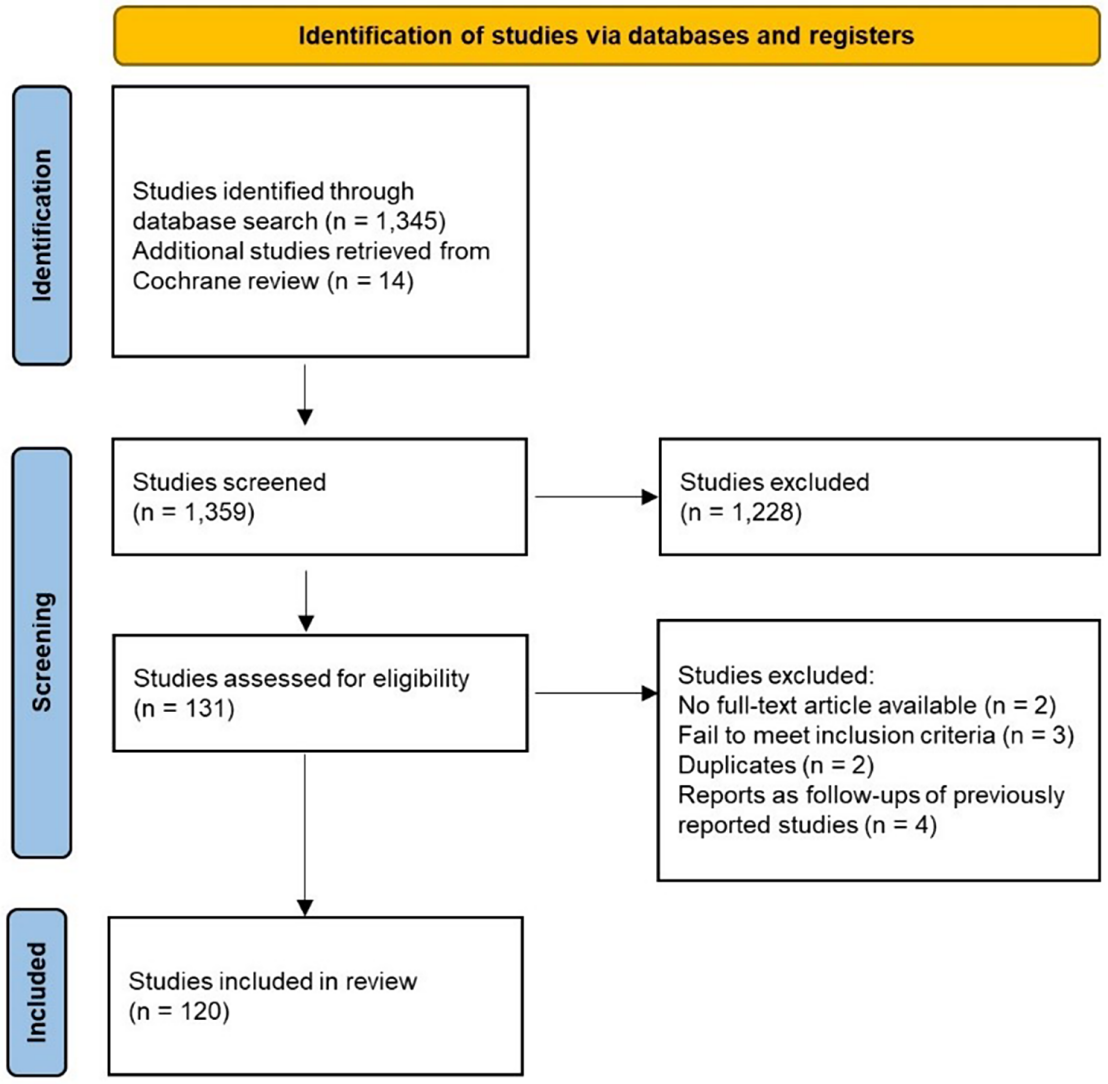
PRISMA systematic review flow diagram according to Page et al. [20].

The literature search resulted in 1,345 titles and abstracts which were identified for screening. In addition, 14 studies which were used for evaluation in the Cochrane review [14], but not identified in our search, were included. From the 1,359 studies, 120 met the eligibility criteria and were included in the review after removal of duplicates and studies for which the full text was not available (Fig. 1).

### Study characteristics

Studies were classified into two types of interventional trials (randomized controlled trials (RCT), and non-randomized interventional studies (classified as interventional in the manuscript and evidence table)) and two observational types (cross-sectional and longitudinal cohort studies (named longitudinal in the manuscript and evidence table)). Details about the characteristics of these study types can be found elsewhere [21]. Most of the studies included in this review were interventional studies, especially RCT (73; 61%) while 14 (12%) were non-randomized interventional studies. From the remaining 33 observational studies, 23 (19%) were longitudinal cohort studies and 10 (8%) were cross-sectional trials. In case of interventional studies (*N* = 87), the investigated product was used ad lib in 72 (83%) studies, 5 (6%) studies had a combination of ad lib and controlled use sessions while a strictly controlled use was applied in 8 (9%) studies. 8 of the 13 studies with a controlled use were PK studies for which a controlled use is quite common to assess nicotine intake and abuse liability [8]. The majority of the studies (92, 77%) investigated ECs while the newer inhalable category of HTP was examined in 20 (17%) cases. 13 (11%) studies investigated non-inhalable products (smokeless (*N* = 1), snus (*N* = 7) or ONP (*N* = 5)). Number of participants varied largely from 11 [22] to 5.1 million in the South Korean nationwide cohort study [23] with a median number of 156.5 participants. Study length differed between one day up to 8 years [24]. Median study length was 3 months (75th percentile: 12 months; 25th percentile: 1 week). The short-term studies were cross-sectional, cross-over PK studies as well as short-term randomized controlled switching trials, while large population-representative cohorts could be investigated over longer time frames. RCT had a median number of 102 participants over 1 month while the non-randomized interventional studies were conducted with a median of 40 participants for 7 months. The number of participants varied extensively as illustrated by the means which differ widely from median values. RCT mean sample size was 275 while interventional trials had 4064 participants on average. In terms of the observational studies, cross-sectional trials had a median number of 181 participants (mean: 577). Obviously, longitudinal cohort studies were conducted over the longest time frame of 24 months and the largest number of participants with a median of 3334 (mean: 5240) compared to the other study types.

Most studies were conducted in the US (48, 40%) followed by the UK (15, 13%), Italy (14, 12%), Japan (9, 8%), and Poland (6, 5%). Only 4 studies were located in LMICs (China [25], India [26], Turkey [27], and one study in several LMICs worldwide evaluated from the Global Adult Tobacco Survey [28]).

With respect to the inclusion criteria, we focused on the people’s use behavior and their willingness to quit smoking during the synthesis of the study data for this review. Most studies recruited people who exclusively smoked 10 or more cigarettes per day (CPD) for 6 months or more. However, there were some trials in which participants were recruited with regular use of only one or more CPD and shorter time frames of daily smoking. The criterion of daily smoking became less stringent in the large longitudinal studies like those evaluating data from the large population assessments, for instance the population assessment of tobacco and health in the US (PATH) or in South Korea (KNHANES) categorizing users into “every day” and “someday” vaping [29] or smoking [30]. The intention (or no intention) to quit or reduce smoking was defined as an inclusion criterion in 59 studies (49%). Sixty studies (50%) included a control group who continuously smoked, while people who never smoked, were rarely comprised as a control group to compare baseline levels, for instance in exposure or health-related measures (16, 13%). Complete quitting (with or without NRT) was reported in 39 studies (35 RCTs, 2 longitudinal, 1 cross-sectional, 1 interventional) as a comparator to switching and former smoking was evaluated in 4 longitudinal and 2 cross-sectional trials as an independent arm/cohort. In contrast, the majority of studies (75, 63%) reported no group or study arm that resembled complete cessation.

Several approaches were used in the different studies to evaluate the products’ potential for helping people to switch to and quit smoking. Use behavior, questionnaire-based assessments (QBA), abstinence, and PK were captured as measures for cessation efficacy in this review. While PK analysis is straight-forward, the other three categories can be evaluated by different assessments as depicted in Table 1. Details regarding the QBA can be found in the respective publications cited in the evidence table. The QBA were stratified into motivational (likelihood of quitting), craving and withdrawal symptoms, and product satisfaction (liking).

**Table 1:** List of measures categorized into QBA assessments, abstinence and use behavior

QBA assessments	Abstinence	Use behavior
Motivational: -11-point Juster probability scale -Time to first CC after waking -Harm perception scale QBA -Intention/Motivation to quit scale -Perceived Health Risk scale (PRIP) Craving & Withdrawal symptoms: -Beck Depression Index (BDI) -Fagerström-Test of nicotine dependence (FTND) -Glover-Nilsson Smoking Behavior (GNSBQ) -Minnesota Nicotine Withdrawal Scale (MNWS) -Craving scale -Modified Cigarette Evaluation Questionnaire (mCEQ) -Visual analogue scales (VAS) -Brief QBA of Smoking Urges (QSU) -Heaviness of Smoking Index (HSI) -Center for Epidemiological Studies -Depression scale (CES-D) -Wisconsin Inventory of Smoking Dependence Motives (WISDM) -Mood and Physical Symptoms Scale (MPSS) -Drug Effects Questionnaire (DEQ) -Physical component summary (PCS) -Mental-component summary (MCS) -Role-social-component summary (RCS) -Penn State Cigarette Dependence Index (PSCDI) Liking / Product satisfaction: -Satisfaction on x-point scale -Quality of Life QBA (QoL) -Single-item attitudinal scale QBA -Liking scale QBA -Visual analogue scales (VAS) -Perception scale (recommendation to friends) -Modified Cigarette Evaluation Questionnaire (mCEQ) -Intent to use questionnaire (ITUQ) -Product Evaluation Scale (PES)	-Short-term abstinence (x-day (point prevalence) abstinence from smoking by self-report) -Long-term abstinence (e.g., 3–6 m (point prevalence) abstinence from smoking by self-report) -No. of quit attempts past x months -Quit rate -Smoking reduction -Relapse rate -Odds ratio: Probability of switching -Smoking resistance task: demonstrate 12-h abstinence from all tobacco	-Cigarettes per day -Puffs per day -Liquid per day -sessions per day -Sticks per day -Pouches per day -total no of puffs -puff duration -inter-puff interval -frequency of use

Biomarkers of exposure (BoE) and biomarkers of potential harm (BoPH) are commonly measured for the risk assessment of the products. Urine and/or blood collection for biomarker analysis can easily be implemented into a smoking cessation trial adding data needed to evaluate the potential for harm reduction after switching. BoE and BoPH were determined in 64 studies (62%), from which 48 were RCTs, 8 interventional, 5 cross-sectional, and 3 longitudinal studies.

The investigated product categories stratified by cessation efficacy measures (details in Table 1) and study types are summarized in Table 2.

**Table 2:** Summary of study types, cessation efficacy measures, number of studies including BoE/BoPH, compliance monitoring and product familiarization per product category

Product / study type (*N*)	QBA / *N* (%)	Abstinence / *N* (%)	Use / *N* (%)	PK / *N* (%)	BoE/BoPH / *N* (%)	Compliance monitoring / *N* (%)	Familiarization / *N* (%)
All (120)	72 (60%)	52 (43%)	97 (81%)	17 (14%)	64 (62%)	77 (64%)	40 (33%)
EC / all (92)	54 (59%)	48 (52%)	78 (85%)	11 (12%)	45 (49%)	60 (65%)	28 (30%)
EC / RCT (52)	31 (60%)	24 (46%)	42 (81%)	9 (17%)	33 (63%)	43 (83%)	18 (35%)
EC / Interventional (14)	12 (86%)	9 (64%)	14 (100%)	2 (14%)	8 (57%)	10 (71%)	10 (71%)
EC / Cross-sectional (9)	3 (33%)	2 (22%)	8 (89%)	NA	4 (44%)	5 (56%)	NA
EC / Longitudinal (17)	8 (47%)	13 (76%)	14 (82%)	NA	0 (0%)	2 (12%)	NA
HTP / all (20)	10 (50%)	3 (15%)	16 (80%)	2 (10%)	14 (70%)	14 (70%)	6 (30%)
HTP / RCT (13)	8 (62%)	1 (8%)	11 (85%)	2 (15%)	12 (92%)	11 (85%)	6 (46%)
HTP / Interventional (0)	-	-	-	-	-	-	-
HTP / Cross-sectional (2)	-	-	2 (100%)	NA	-	1 (50%)	NA
HTP / Longitudinal (5)	2 (40%)	2 (40%)	3 (60%)	NA	2 (40%)	2 (40%)	NA
Snus, Pouch / all (13)	10 (77%)	2 (15%)	8 (62%)	4 (31%)	6 (46%)	7 (54%)	8 (62%)
Snus, Pouch / RCT (9)	9 (100%)	1 (11%)	6 (67%)	4 (44%)	4 (44%)	5 (56%)	8 (89%)
Snus, Pouch / Interventional (0)	-	-	-	-	-	-	-
Snus, Pouch / Cross-sectional (2)	1 (50%)	-	1 (50%)	NA	1 (50%)	2 (100%)	NA
Snus, Pouch / Longitudinal (2)	-	1 (50%)	1 (50%)	NA	1 (50%)	-	NA
NA: not applicable							

### Compliance measures and familiarization

Biochemical verification of the participants’ product use status during the study was applied in the majority of the studies (77, 64%). The use status was monitored during screening but also over the course of the study in long-term trials where the participants returned to the clinic at specified intervals for biospecimen collection and clinical assessments. Yet, compliance monitoring over time was not included in all long-term studies. 10 of the 14 non-randomized interventional studies comprised compliance monitoring at screening and during the course of the study. The longitudinal cohort studies mostly relied on stratification into exclusive (but also dual) use by self-report with only 4/23 studies having a biochemical verification of the use status. In RCTs, the product use compliance was frequently verified during screening, with no further monitoring in the controlled short-term trials. Yet, for two studies with longer trial periods, no compliance monitoring after screening was reported [31, 32]. Exhaled carbon monoxide (CO) is by far the most frequently used biomarker for verification of the smoking status, sometimes accompanied by additional BoE, especially cotinine in urine or saliva. Exhaled CO was monitored in 64 of the 77 studies (83%) where compliance was measured with varying thresholds between 4 and 15 ppm. Cotinine in urine or saliva was determined in 32 studies (42%) to verify smoking status at 200 ng/mL or 500 ng/mL in most cases. Other BoE used for compliance were NNAL, anabasine (AB), 3-trans-hydroxycotinine (OH-Cot) and the cyanoethylvalin Hb adduct (CEVal), each of them used in one study in combination with CO (NNAL) [33], or CO and cotinine (AB, OH-Cot, CEVal) [34–36]. One study applied the long-term biomarker of acrylonitrile exposure CEVal together with AB and anatabine (AT) [37]. However, none of the studies applied objective measures to verify the EC use status by appropriate biomarkers of compliance. Trials with an intervention to a test product comprised a familiarization period in 40 (46%) of the interventional studies, meaning that participants had no opportunity to familiarize with the product in more than half of the trials.

### Summary of the characteristics of the studies from the Cochrane review on cessation efficacy of ECs

39 studies which were included in the meta-analysis in the recent Cochrane review [14] regarding the efficacy of ECs for smoking cessation were evaluated here [27, 32, 33, 35, 36, 38–71]. As the Cochrane review concluded with high certainty evidence on the cessation efficacy of ECs, we decided to determine the study characteristics of those 39 studies specifically as depicted in Table 3.

**Table 3:** Study characteristics of the 39 studies which were identified as eligible for the Cochrane review on the cessation efficacy of ECs [14]

Sample size	Country	Study type	Study duration	Control groups	Compliance measure	Familiarization	Cessation efficacy measures	BoE/BoPH
Median: 71 Mean: 226 Min-Max: 12-1563	US: 14 Italy: 8 UK: 7 Australia, Greece: 2 Belgium, Canada, New Zealand, Poland, South Korea, Turkey: 1	RCT: 30 Interventional: 9 Cross-sectional: 0 Longitudinal: 0	Median: 4 m Min-Max: 0.4–24 m	CC: 13 NS: 0 Quitters: 15	CO: 25 CO/Cot: 4 Cot: 1 CO/Cot/AB: 1 CO/Cot/ OHCot: 1 CO/NNAL: 1	16	QBA: 24 Abstinence: 24 Use: 36 PK: 3	28

AB: anabasine; BoE: biomarker of exposure; BoPH: biomarker of potential harm; CC: consumer of combustible cigarettes; CO: carbon monoxide; Cot: cotinine; m: months; Max: maximum; Min: minimum; NNAL: 4-(methylnitrosamino)-1-(3-pyridyl)-1-butanol; NS: never smoker; OH-Cot: trans-3’-hydroxycotinine; PK: Pharmacokinetics of nicotine; QBA: Questionnaire-based assessments; RCT: randomized controlled trial

All studies comprised an intervention with an EC. Hence, only RCT (77%) and non-randomized interventional studies (23%) were included but no longitudinal cohort or cross-sectional studies. In comparison to all 120 studies included in our review, the median sample size was lower (71 versus 156.5) while the median study duration was moderately longer (4 versus 3 months). Only one third of the 39 studies included a positive control group of people who continue to smoke, while an abstinence group of people who quit without an EC was assessed in 15 (38%) studies which is in the same percentage as for the whole sample (45, 37%). The percentage of the different cessation efficacy measures applied in the studies of the Cochrane review generally resemble those of the whole set of studies. Notably, abstinence evaluation was more frequent in the “Cochrane studies” (64%) compared to 43% for all studies. Familiarization was implemented into the study design in 16 (41%) of the studies which is consistent to the complete set of interventional studies (46% use of a familiarization period). 33 of the 39 studies verified the compliance, mostly by exhaled CO alone or in combination with other BoEs like cotinine or NNAL.

## Discussion

### Best practice and limitations in the study designs to assess the cessation efficacy and harm reduction potential of nicotine products

In order to address the efficacy of a product to help people quit smoking, several measures have been applied which were divided into the three categories of QBA, abstinence and use behavior. In addition, PK is an important measure to determine if delivery of nicotine follows similar kinetics to those of smoking in order to suppress the cravings and withdrawal symptoms. The QBAs used in the studies can be categorized into such addressing motivational aspects, cravings and withdrawal symptoms as well as product satisfaction. In combination with PK, these assessments are meant to reveal the abuse liability of the products. Assessing the abuse liability comprehensively is important to decipher the potential of the product to transition people who smoke completely away from cigarettes [8].

Abstinence can be either assessed as short-term (e.g., 7-day point prevalence) or continuous abstinence over several months. The number of quit attempts or its counterpart, the relapse rate, during the course of a study can give additional information regarding the cessation efficacy of a substitute product. These measures clearly demonstrate the efficacy of a cessation treatment which should be verified biochemically by suitable compliance markers. Product use per day gives a quantitative measure for the consumption of a product and shall always be determined. It has to be emphasized that these measures were all based on self-report despite the short-term trials under confinement which are usually no longer than one week or two which clearly is not sufficient to robustly detect cessation efficacy. This is where compliance monitoring comes into play, since it is mostly the RCTs, non-randomized interventional trials but also observational longitudinal studies with a study (or observation) period of several months up to years which give meaningful results in terms of efficacy. A robust and sensitive verification of the participants’ use compliance is important in order to receive reliable data for product use and abstinence rates. Exhaled CO has been mostly used for this purpose followed by cotinine in urine. Both biomarkers come with several limitations. Exhaled CO is significantly elevated in people who smoke at 2–18 ppm in contrast to people who do not smoke with 1–4 ppm [72]. Yet, regarding the overlap in exhaled CO levels, the set thresholds in the studies between 4 and 15 ppm appear somewhat arbitrary. Furthermore, exhaled CO has a short half-life of around 4.6 ± 1.6 h meaning that it can only detect very recent smoking [73]. In addition, it is insensitive and light smoking and/or shallow inhalation may not be detected accurately [73, 74]. Cotinine as the major metabolite of nicotine is not specific to smoking as it will be detected by exposure from any nicotine containing product making it obsolete in long-term switching trials. The use of a biomarker specific to smoking and with a longer half-life would be desirable in combination with biomarkers specific to the use of other nicotine products such as ECs or ONPs. Our suggestions regarding suitable biomarkers are summarized in the section “Points to consider in the study design addressing the cessation efficacy of new tobacco and nicotine products”. For a comprehensive discussion about this topic refer to our recent review [75].

In terms of QBA, there is a large variety of questionnaires applied to determine the addictiveness and the attractiveness of the products specifically addressing addiction, craving, withdrawal symptoms as well as product satisfaction. Most of the QBAs use a scale, often times visual analogue scale (VAS), which is helpful in the correlation with PK and use data (e.g., CPD) or other numerical data. The difference in the product types may indicate the need for the adaptation of the QBA as done for the cigarette evaluation scale which has been adapted for ECs, HTPs and oral products [33, 71, 76–80]. Combining suitable QBAs, measures for abstinence and use behavior with a nicotine PK would be a desirable strategy for a comprehensive investigation of the abuse liability of the product. Yet, only 6 out of the 120 studies combined QBA, use behavior and PK, predominantly in order to determine the product satisfaction but not specifically the efficacy for cessation. Nicotine PK in people who stop smoking (who only use the alternative product), people who reduce smoking and use the alternative product and those who relapsed (returned to smoking CC only) may give new insights into the product characteristics which are favorable for quitting smoking. The nicotine PK can be assessed during and after an *ad lib* product use session of the respective alternative product. It is hypothesized that a steep increase in nicotine blood levels at concentrations comparable to cigarette use is more likely to satisfy nicotine cravings and hence facilitate switching and completely quitting CCs [8]. Comparing the pharmacokinetics of people who stopped smoking, those who smoke and use an alternative and those who relapsed to smoking can help to identify PK parameters, e.g., the PK profile, maximum concentration (C_max_), time of maximum concentration (T_max_), and area under the curve (AUC), predictive of the individual’s cessation rate.

A main outcome for the Cochrane review was quitting/abstinence for at least 6 months [14]. Yet, the majority of the long-term studies relied on ambulatory visits and online-based surveys during the course of a study. With the high availability of smartphones and internet worldwide including in LMICs, app-based QBAs and use recording can facilitate documentation making the data accessible in real time for future studies as demonstrated in other fields [81–83]. Especially product use and abstinence from smoking could easily be recorded on a daily basis. Moreover, product evaluation scales could be monitored periodically to correlate product liking with use and quit rates. However, participants should not be overwhelmed by the amount and frequency of questions. Regarding daily questions, participants must be able to answer in a fast and simple manner. For instance, a preselection of answers may improve the response rates as exemplified here for the question to record daily use: “How many cigarettes did you smoke yesterday?” with a response selection: e.g., 0, 1–5, 6–10, 11–15, 15+. From our experience, this way, it is more likely that participants will respond on a daily basis. Recording this question gives information on product use and abstinence from smoking at the same time.

Another limitation revealed by our review was a lack in the characterization of the participants regarding their motivation to quit and their use status. Willingness to quit or reduce smoking may have an impact on quitting rates. The same applies for the motivation to try a new product. However, 51% of the studies did not report the motivation of the participants, let alone defining the motivation to switch or intention to quit as an inclusion criterion. 26 studies included participants motivated to quit while those not willing to quit were recruited in 33 studies. Complete cessation in people who switch to an alternative product, although they are not intending to change their smoking behavior, may yield stronger evidence about the product’s potential to serve as a smoking cessation aid. It is therefore appropriate to define participants’ willingness/intention to quit smoking as an inclusion (or exclusion) criterion or at least describe why this characteristic was not considered for recruitment. Moreover, the product use status was poorly defined especially in the observational longitudinal studies. A clear definition of the use frequency (e.g., daily or number of days per month) and discrimination between use groups is very important for an accurate interpretation of abstinence rates. For instance, if smoking is only defined by taking a puff no matter if daily or some days, lower cessation rates will be concluded compared to a stricter discrimination between use states, e.g. daily use and correlation with CPD. A product familiarization period was only reported in 46% of interventional studies. This becomes critical especially when participants who have no experience with a product are included. It is known that people who smoke show a different use pattern when they initiate EC use compared to experienced EC users [84, 85]. Moreover, the individual preferences in terms of flavors and nicotine strengths can be crucial for the outcome of a cessation/switching efficacy study. If study participants are limited to one product they do not like while there may be a flavor variant they would like, this will quite probably have an impact on their quit rates. Thus, a thoughtful consideration of product choices accompanied by a familiarization period at the beginning of the study may better reflect the products’ cessation efficacy and result in more realistic quit rates. Finally, only 3% of the included studies were conducted in LMICs indicating a huge bias towards Western countries. In our view, this bias needs to be tackled in future research, as cessation efficacy of a product category can differ between regions due to varying use prevalence and cultural habits [86, 87]. For instance, oral tobacco use is very popular in South Asia [86] meaning that ONPs may be a more appealing alternative compared to ECs or HTPs. In contrast, Japan is a technology affine high-income country where consumers of CCs switched to HTP (note that ECs are illegal on the Japanese market) rapidly after market introduction [88]. It has to be emphasized that our review focused on the identification of methodological limitations in order to make researchers aware of common pitfalls to avoid. While beyond the scope of this review, it would be interesting to elaborate the impact of the weaknesses on the results, meaning to which extent do these design failures cause false results? Moreover, confirmation or reporting bias can occur by means of the authors’ competing interests like industry or philanthropic funding, which is a general ethical issue extensively discussed in many disciplines not only in tobacco science [89–92].

### Points to consider in the study design addressing the cessation efficacy of new tobacco and nicotine products

The limitations in numerous studies and the characteristics of the study designs which were included into the Cochrane review guided the recommendations for the main properties of clinical studies with the objective to determine the cessation efficacy by switching to a new nicotine product.

Evidently, the trial shall be interventional, either an RCT or non-randomized longitudinal study measuring abuse liability by QBA(s) applicable to the product under investigation, abstinence, use behavior and nicotine PK. Participants’ motivation to quit and switch shall be defined as an inclusion criterion as well as a threshold for use per day and duration of cigarette use at baseline. Ideally, participants shall be allowed to use more than one nicotine product for smoking cessation to improve the chance of quitting smoking altogether by a complete switch. Moreover, a control group of people who quit using NRT instead of the investigated product shall be included to compare the abstinence rates. It has to be emphasized that due to the heterogeneity of the products, the switching rate of one study product cannot resemble the cessation efficacy of the whole product category. However, given the lack of studies providing a variety of products and missing data with respect to an appropriate familiarization period, objective recommendations regarding those parameters cannot be deduced. Use of different products must be accurately recorded throughout the study, which can easily be done for instance app-based via peoples’ smartphones. Abstinence from smoking should be verified by an appropriate BoE. CEVal, the globin adduct of acrylonitrile, could be considered due to its long persistence in blood after exposure (long-term compliance marker) and its specificity for smoking [93]. Other BoE may be relevant to monitor product use compliance depending on the product type, for example anabasine/anatabine for smokeless tobacco users [37] and propylene glycol for vapers of ECs [94, 95], respectively. In case of budgetary constraints, the investigators may need to compromise the monitoring frequency. Measuring CEVal only monthly or every two months would still yield valid information about the participants use behavior taking into account its long-term detectability. Alternatively, the urinary mercapturic acid metabolite of acrylonitrile – 2CyEMA or CEMA – can be measured which does not require blood sampling. Robust cut-offs of 7.32 ng/mL and 11.4 µg/g creatinine for distinguishing cigarette smoking from not smoking were set for 2CyEMA based on PATH study data [96]. But, 2CyEMA comes with the limitation of a much shorter half-life of 7–9 h [97].

The implications for harm reduction by switching can be combined with the outcome on cessation in such a study setting. In order to assess the risk reduction, it is recommended to analyze suitable biomarkers of exposure and potential harm. A guideline towards relevant BoE [75] and BoPH [98] depending on the research question can be found elsewhere.

The mean study duration of the 39 studies included in the Cochrane review was 5.3 months (median 4.0 months). Excluding the shorter-term trials (≤ 1 month), a mean study length of 7.4 months (median: 6 months) was obtained. Hence, the study duration shall be at least 6 months while an estimate of the number of participants cannot be generalized. For orientation, the studies from the Cochrane review with a study duration of 6 or more months (*N* = 19) averaged 330 participants (median: 150). Yet, the size highly depends on the study design and length as well as the product type, as these characteristics trigger cessation rates. Moreover, the primary endpoints influence the study size which is needed for sufficient statistical power. Low number of participants of 102 as averaged in RCTs may not be sufficient for a conclusive statement on a product’s efficacy. The appropriate sample size needs to be assessed as part of the statistical analysis plan (SAP) of the study and we urge researchers to publish the SAP together with the study protocol for the sake of transparency and good research practice.

## Conclusions

In conclusion, the discussed points for consideration shall help researchers to avoid common flaws and improve the quality of the studies for a more robust investigation of the cessation efficacy by switching to an alternative reduced risk product or NRT. We are aware that not all of the measures recommended here can be applied due to budgetary, time or technical constraints. Yet, we encourage researchers to stay aware of the limitations that can derive from omitting some of the aforementioned design features and the potential effects on interpretation of their study results.

### Electronic supplementary material

Below is the link to the electronic supplementary material.

Supplementary Material 1

Supplementary Material 2

## Data Availability

The datasets used and/or analysed during the current study are available from the corresponding author on reasonable request.
