# Supplementary material for: The potential of new nicotine and tobacco products as tools for people who smoke to quit combustible cigarettes – a systematic review of common practices and guidance towards a robust study protocol to measure cessation efficacy
Source: Harm Reduct J. 2024 Jul 5;21:130. doi: 10.1186/s12954-024-01047-1 (PMC11225172; doi:10.1186/s12954-024-01047-1)
Supplement: Supplementary file 1 — Supplementary Material 1 [file 12954_2024_1047_MOESM1_ESM.docx]

*SUPPLEMENTARY INFORMATION to*

**The potential of new nicotine and tobacco products as tools for people who smoke to quit combustible cigarettes – A systematic review of common practices and guidance towards a robust study protocol to measure cessation efficacy**

Nikola Pluym*, Therese Burkhardt, Gerhard Scherer, Max Scherer

ABF Analytisch-Biologisches Forschungslabor GmbH, Semmelweisstr. 5, 82152 Planegg, Germany

^*^ Corresponding author:
Nikola Pluym ABF Analytisch-Biologisches Forschungslabor GmbH, Semmelweisstr. 5, 82152 Planegg, Germany

E-Mail: [nikola.pluym@abf-lab.com](mailto:nikola.pluym@abf-lab.com); Tel.: +49 89 411479612

**Search terms and filters**

Pubmed search:

(smok* OR tobacco) AND (quit* OR switch* OR reduction) NOT low-nicotine NOT socioeconomic* [tiab] NOT varenicline [tiab] NOT bupropion [tiab] NOT covid [tiab] NOT HIV [tiab] NOT diabet* [tiab] NOT schizophren* [tiab] NOT depress* [tiab] NOT homeless [tiab] NOT alcohol+abuse [tiab]

Filter:

((e*cigarette* OR electronic nicotine delivery OR heated tobacco OR tobacco heated product OR snus OR oral nicotine OR oral tobacco OR smokeless tobacco OR use behavior) AND (healthy OR Randomized Controlled Trial OR Clinical Trial OR longitudinal trial OR Prospective trial OR prospective stud* OR randomized controlled study OR clinical stud* OR long-term stud* OR long-term trial OR cross-sectional stud* OR ambulatory study OR prospective cohort study) AND (Nicotine OR tobacco OR Cigarette) NOT dental NOT animal NOT psycholog* NOT pregnan* NOT youth) NOT (Review [publication type] OR Systematic review [publication type])

Additional filters:

Abstract, Humans, English, MEDLINE, 2014-2022

Table S1: Detailed evidence table

| Reference | Participants (N) | Region / Country | Study Type / Study design | Study length | Intervention & Products evaluated | Compliance monitoring (Y/N) | familiarization  (Y/N) | Endpoints for efficacy and use behavior | Endpoints for risk/exposure |
| --- | --- | --- | --- | --- | --- | --- | --- | --- | --- |
| Adriaens 2014 ^1^ | 48 S (EC naïve) no intention to quit  27f / 21m | Leuven area / Belgium | RCT with 3 use sessions over 8 weeks  Interventional: EC use in 3 sessions 5min EC product use and free ad lib use within the 8 weeks  Control: CC smokers  Inclusion: S of 10CPD >3years with no intention to quit | 8 weeks | Study EC  EC vs CC  Choice of 2 2^nd^ gen ECs | Y: eCO | N | QA: Craving & withdrawal symptoms (FT, BDI); Smoking reduction and  Quit rate at end of study and follow-up (3 months) | eCO  Cot (saliva) |
| Azagba 2020 ^2^ | 20,558 | USA | PATH Wave 1 (2013/14) and Wave 2 (2015/16)  Longitudinal: EC use at W1 and change in smoking frequency between W1 and W2  Classification of S: Former / Experimental (< 100 C lifetime) / Every day / some day based on 3 questions  EC use classification based on 4 questions: Every day / some day / Experimental (never used EC fairly regularly and current every day or someday use) | 2 years | No intervention Observational (Longitudinal)  EC | N | n/a | Use behavior: Smoking / Vaping frequency -> Association of EC use with transition in smoking behavior (odds ratio) | none |
| Azzopardi 2022a ^3^ | 34 experienced S > 10CPD for 6months | Montreal, Canada | Single-center, RCT, 3-product, 3-period, crossover, single dose PK study  Washout period of 24h. Each of the 3 products per subject with 24h washout in between.  S > 10CPD for >6m willing to abstain from CC (intention to quit was not a criterion) | 5 days | Study product: Nicotine pouch and reference nicotine gum and lozenge | Y: urine cot test | Y: Training sessions in clinic: each of products consumed once | QA: Subject effects of product use: e.g. satisfaction; taste; joy of sensation in mouth in a 7-point scale | Nicotine PK evaluation |
| Azzopardi 2022b ^4^ | 220 (total):  100 experienced pouch users and 40 per group of current S, former S and NS | Denmark  Sweden | Cross-sectional confinement for 24h  Use of usual product (pouch or CC)  Pouch and CC use confirmed by urine cot test and eCO (Cot + / eCO +: S; Cot + / eCO-: Pouch)  Control: S (>12m smoking), former S and NS  Experienced NP users (>6m pouch use) | 24h | No intervention / Observational (Cross-sectional): Experienced pouch users and smokers (< 6m pouch use and < 12m CC) | Y: **CeVal** for long-term abstinence in Pouch group  **AB/AT:** short-term abstinence from smokeless tobacco | Experienced users only | Quality of life assessment: 36-Item Short Form Health Survey questionnaire (RAND Corporation)  URL: <https://www.rand.org/health-care/surveys_tools/mos/36-item-short-form/survey-instrument.html> | BoE urine: 24h urine  NNAL, NNN  TNE  MHBMA, HMPMA, 3-HPMA, SPMA  3-OH-BaP  COHb  BoPH:  HDL, Eicos (8iPGF, 11-dhTXB2)  sICAM-1  carotid intima-media thickness (CIMT)  FEV1, FENO, WBC |
| Baldassari 2017 ^5^ | 40 Smokers | Yale / USA | RCT 1:1 randomization into nic and non-nic ECs  Smokers with intention to quit  Intervention with EC  Inclusion: 1+CPD | 8 weeks + 6 month follow-up | Nic EC and non-Nic EC  Both groups received nic patch  2^nd^ gen EC tank system; e-liquid 0 or 24 mg/mL Nic) | Y: eCO (6 ppm) | N | EC use if patch was not sufficient for cravings. Endpoint: CPD @ week 8 and week 24 | FEV1  FVC  Spirometry  FeNO |
| Bell 2017 ^6^ | 30 Smokers with HIV | Brisbane Australia | RCT in Smokers switching to study EC  Continuous provision of e-liquid and monitoring of consumption  Inclusion: >5CPD; S for >12m with no intention to quit | 6 months | Study EC: Innokin Endura (Tanksystem) with 12mg/mL Nic | N | N | CPD  Quit attempts  Short-term (7-day) abstinence from CC smoking @ weeks 4, 8, 12, 24  Medium-term (> 8 weeks) abstinence from CC smoking @ weeks 12, 24  FT  Glover Nilsson Smoking Behavioral QA (GNSBQ) | none |
| Berenguer 2022 ^7^ | 10 Smokers + 8 EC vapers | Funchal / Portugal | Cross-sectional  Control: Non-smokers  Inclusion: Healthy | 1 day | Observational (Cross-sectional) No intervention  Cross-sec  EC vs CC | N | N (cross-sectional): Use of UB | none | none |
| Blank 2019 ^8^ | 11 Dual Users (CC + EC) | Dunedin / New Zealand | Feasibility study  Interventional cessation study  S (Daily S) intention to quit and willing to use EC for quitting  Unregular Use of EC for quitting within 3months before study  CPD: 5 – 20  No controls | 8 weeks | EC (vape pen) with own liquid with smartphone App | N | Y: Training session for study EC | App-based recording of voltage (EC use) on participants smartphone  Daily survey via text massage to report CPD and EC use (puffs/d and sessions/d)  Time to first CC after waking  **11-Point Juster probability scale**: Scale 0-10 for likelihood of quitting with help of EC and confidence of quitting in next month | none |
| Burris 2014 ^9^ | 57 S | S.Carolina USA | Pilot study / RCT  Healthy S with no intention to quit  >10 CPD over last 12 months  Control group: Continue S | 2 weeks | Smokeless Camel Snus Pouch | N | Y: 3 day trial period | CPD  Quit attempts  FT for Nic dependence  Craving scale QA  Single-item attitudinal scale QA  Liking scale QA  Harm perception scale QA  Motivation: Intention to quit scale | Cotinine  CO_ex_ |
| Camacho 2014 ^10^ | 301 S | Hamburg Germany | RCT switching study to RTP CC after 2 weeks in study for 4 more weeks. Switch to study product after 2 weeks of CC for 4 weeks.  Inclusion: 10-30 CPD; S for > 3 years  Control: CC continue to smoke + NS | 6 weeks | RTP Cigarettes | N | N | None | 21 BoE in 24h urine |
| Caponnetto 2013 ^11^ | 14 S | Catania Italy | Interventional switching study in 14 Smokers with chronic schizophrenia  Inclusion: >20CPD for >10 years no intention to quit  Control: none  6 visits (BL; 1m, 2m, 3m, 6m, 12m) | 12 months | Study EC ad lib use | Y: eCO (10ppm) | N: only use instruction | CPD  Smoking abstinence/Quit rates  EC use | eCO |
| Caponnetto 2013 ^12^ | 300 S | Catania Italy | 3-arm double-blind RCT in Smokers no intention to quit switching to EC for 12 weeks and follow-up after 12 months  Intervention phase of 12 weeks and observational phase thereafter. 9 visits (BL, 2w, 4w, 6w, 8w, 10w, 12w; 24w, 52w)  Inclusion: Healthy S >10 CPD for >5years with no intention to quit; Control: non Nic EC | 12 months | 1^st^ gen EC in 2 different nic strengths and without nic | Y: eCO (7ppm) |  | CPD  Smoking abstinence/Quit rates  Visual analogue scale (VAS) to assess perception and liking  EC use  Perception of EC scale: Recommend to friends | eCO  Cotinine (saliva)  Dry cough  Mouth / throat irritation  Shortness of breath  Headache |
| Caponnetto 2020 ^13^ | 220 S | Catania Italy | 12 weeks single-center RCT with healthy smokers with follow-up after 24 weeks. 7 visits (BL, 1w, 2w, 4w, 8w, 12w) and 24w follow-up  Inclusion: Healthy S >10CPD >1year with no intention to quit were randomized to HTP or EC (1:1)  Control: No control | 12 weeks | HTP or EC  HTP vs EC for cessation  HTP: IQOS  EC: tank system “JustFog” | Y: eCO (10ppm) | Y: Training & counseling  Try and choose flavors | Smoking abstinence (self-report)  CPD  Use of investigational product (HTP stick/d or EC ml e-liq/d)  Modified Cigarette Evaluation Questionnaire (mCEQ) adapted for EC and HTP users  Three of 8 items of “Smoking Cue Appeal” adapted for EC and HTP users  Intent to use questionnaire (ITUQ) – items 4–6; PRI-P CC Perceived Health Risk scale (for classic cigarette);  PRI-P RRP Perceived Health Risk scale (for reduced risk products) | Chester Step Test for VO2max  Quality of Life QA  BoE Urine:  NNAL  HEMA, MHBMA, HMPMA, 3‑HHPMA, SPMA, AAMA, GAMA, CEMA, 2-HPMA  OH-PAHs (Nap, Flu, Phe, Pyr)  Cot |
| Caponnetto 2018 ^14^ | 12 S | Catania Italy | RCT with 12 healthy S  Inclusion: healthy S >10CPD for >5 years  Control: CC | 1 day | HTP controlled use  2 HTPs: IQOS & Glo | Y: eCO (10ppm) | Y: Training for >30min | None | eCO |
| Carpenter 2017 ^15^ | 68 S | S.Carolina USA | RCT with S with minimal (if any) EC history randomized to EC  Stratification of S by motivation to quit on VAS scale with 2:1 proportion EC:CC  3 w sampling period allowed to use EC. No further product offered after 3w and follow-up up to 4m  Visits: BL, 2w, 3w, 4w; Follow-up visits: 8w, 12w, 16w  Inclusion: S >5CPD for >1year with no intention to quit  Control: Continue CC | 4 months | EC BluCig  disposable 16mg/mL Nic with menthol or tobacco flavor  EC vs CC | Y: eCO (6ppm)  NNAL | N | **Ecological Momentary Assessment (EMA)** E-mail sent survey 3x per day  Modified Cigarette Evaluation Scale (mCEQ) adapted for EC  CPD  EC use  Quit attempts  Smoking Abstinence  Motivation to Quit (MTQ) by VAS | BoE (Urine)  Cotinine |
| Chapman 2022 ^16^ | 24 DU of CC/Snus | Sweden | RCT cross-over design PK study in DU of CC/Snus; Controlled use under confinement; 1 Use session per day, total of 5 Days/Sessions  Inclusion: DU for > 1year of 2+ snus cans and 5 CC per week no intention to quit  Control: CC PK | 5 days | Nic pouch and CC (comparator)  3 Nicotine pouches  ZoneX in 2 nic strengths and Skruf Snus | N | Y: Only training on use but no product trial | Product Evaluation Scale (PES) for urge to smoke and liking  Visual Analogue Scale (VAS) for urge to smoke & liking | Nicotine PK evaluation |
| Choi 2021 ^17^ | 5.1 Mio Men | S. Korea Nationwide | Nationwide cohort study  Longitudinal study  All participants were divided into continual CC-only smokers, CC and NNTP users, recent (<5 years) CC quitters without NNTP use, recent CC quitters with NNTP use, long-term (≥5 years) CC quitters without NNTP use, long-term CC quitters with NNTP use, and never smokers. Propensity score matching analysis was conducted to further compare CVD risk among CC quitters according to NNTP use. Starting from the second health screening date, participants were followed up until the date of CVD event, death, or December 31, 2019, whichever came earliest. | 5 years | No intervention  Observational (Longitudinal)  EC and HTP | N: Self-report only | n/a | None | CVD: hospitalization ≥2days for coronary heart disease or stroke  Mortality  Lung cancer  COPD |
| Cobb 2021 ^18^ | 520 S | Hershey, PA and Richmond, VA  USA | four-arm, parallel-group, RCT at two sites with exclusive S interested in reducing CC but not quitting  Inclusion: S: > 9CPD >1 year no intention to quit  Provision of study EC (different strength in Nic depending on group and flavor choice tobacco or menthol) for ad lib use to reduce CC use  CC reduction instructed to be 50% in 0-2w, 75% w3-w8 and maintain or further reduce until w24  Complete cessation in follow-up was advised  Compliance not incentivized  9 visits in investigational phase at BL, 1w, 2w, 4w, 8w, 12w, 16w, 20w, 24w and 2 follow-up visits at 28w and 36w  Control: cigarette-shaped tube as CC substitute (no delivery of Nic or aerosol) | 6 months + 3 months follow-up | EC pen-style (cartomiser): eGo-style with 0, 8 or 36 mg/mL Nic vs “Placebo” (CC substitute) | Y: eCO (>9ppm) | N: Only choice between 2 Liquid flavors at beginning (tobacco or menthol) | CPD  EC use | eCO  BoE (Urine)  NNAL  Cot  BoPH (Urine): 8-i-PGF2a  Glutathione  Pulmonary function test |
| Cohen 2021 ^19^ | 300 S | USA (5 sites) | RCT under 8day confinement  Randomization into 6 different JUUL study product types, 1 DU group (DU group with over 50% reduction in CPD to baseline)  6 day ad lib use of study EC  Inclusion: > 10CPD >1year with no intention to quit  Controls: Continue UB CC use and abstinence | 8days | EC JUUL in 4 flavors and 2 nic strengths (50 and 30 mg/mL)  EC vs DU / CC | Y: eCO (<10ppm) and Cot (>200ng/mL) at screening | Y: 30 minute product trial with all 4 flavors with 50mg/mL Nic strength at screening | EC use and CPD | BoE (Urine)  Nic+5 (TNE)  NNAL/NNN  MAs (3-HPMA, MHBMA, SPMA, HMPMA, CEMA)  1-OH-Pyr  AA (o-tol, 2-AN, 4-ABP)  BoE (Blood) COHb |
| Cravo 2016 ^20^ | 420 S | UK (2 sites) | RCT parallel group study to evaluate the safety profile of an electronic vapour product over 12 weeks  Ambulatory design  8 visits (BL, weeks 1, 2, 4, 6, 8, 10, 12) and no follow-up  Inclusion: 5-30 CPD >1 year with no intention to quit S  Control: Continue CC | 3 months | Study EC Prototype EC Fontem Ventures  EC vs CC | Y: eCO (8ppm) + self-report: compliant if no CC smoked for 80% of study days | Y: training at baseline visit | MWS-R scores (revised Minnesota Nic Withdrawal Scale): The questionnaire was modified to include only the 15 questions of subject's part. The core total scores (sum of the first nine questions on behaviour) and the extended total scores (sum of all 15 questions) were calculated. Symptoms (e.g. angry, irritable, frustrated, depressed, restless, insomnia) were rated from 0 (none) to 4 (severe). Extended total scores may range from 0 to a maximum of 60.  QSU-brief scores (Brief QA of Smoking Urges): Ten statements such as “I have a desire for a cigarette right now”, were rated by a number ranging from 1 (strongly disagree) to 7 (strongly agree). Factor 1 scores (sum of questions 1, 3, 6, 7, and 10 for desire and intention to smoke), Factor 2 scores (sum of questions 2, 4, 5, 8, and 9, for anticipation of relief from negative effects with urgent desire to smoke) and total scores (sum of all questions) were calculated. Total scores may range from 0 to a maximum of 70.  CPD  EC use | Vital signs  12-lead ECG  FVC, FEF_25-75_, Peak expiratoy flow (PEF), FEV1  WBC, RBC, Hb, haematocrit (PCV), mean cell volume (MCV), mean cell haemoglobin (MCH), mean cell haemoglobin concentration (MCHC), platelet count, differential WBC  Clinical biochemistry  Urinalysis  BoE (Urine)  Nic+5 (TNE)  SPMA, 3-HPMA, PG, NNAL  eCO, COHb  BoPH  Hb, PCV, RBC, WBC, Cholesterol |
| Czoli 2019 ^21^ | 48 DU | Ontario Canada | Controlled, interventional trial with crossover design in DU. Three consecutive 7-day periods in which the use of CC and EC was experimentally manipulated, resulting in four study conditions (each for 7d): DU, CC, EC, and abstinence. All use of UB CC/EC  DU were forced to switch to exclusive use of CC, EC, abstinence  Ambulatory study  4 Visits: BL and after each 7d period (BL, weeks 1, 2, 3)  Inclusion: ≥5CPD and daily EC use for at least last 7 d with no intention to quit CC  Control: Crossover comparison: CC and abstinence as control conditions | 3 weeks | UB EC Tank-system and CC | Y: eCO and Cot (urine) | N: Use of UB | FT Dependence for CC also adapted for EC  Product use QA  CPD  EC use  Time to first use | BoE:  NNAL, 1-OH-Pyr, Cot |
| D’Ruiz 2015 ^22^ | 24 S | Nebraska  USA | RCT 6-period crossover Nic PK  30 min controlled and 1h ad lib use sessions  Stationary  Product use / PK in-clinic on Days 1, 3, 5, 7, 9, 11  Products must be used all 11 d in clinic  Inclusion: ≥10CPD >1year  No control arms | 11 days | 5 ECs (different flavors) in 16 and 24 mg/mL Nic strength  Cartomizer EC tank system | Y: eCO (>10ppm) and Cot (>500ng/mL) at screening | Y: Instructions and practicing. Demonstrate appropriate use to clinical staff. Provision of product for use prior to study start | FT  Smoking urge by VAS | Nic PK |
| D’Ruiz 2017 ^23^ | 105 S | Nebraska  USA | RCT Switching study to 1 of 6 EC following ad lib use of study EC. Randomization into exclusive EC groups and DU groups  DU group required to reduce CPD by 50% in study  Controlled study in-clinic  Inclusion: ≥10CPD >1year  Control: Abstinence | 5 days | Study EC Blu (Fontem) rechargable and disposable variant in tobacco + cherry flavor  EC vs CC  DU vs CC | Y: eCO (>12ppm) and Cot (>500ng/mL) at screening | Y: only training | CPD (Reduction in DU group)  EC use | Cardiovascular effects: blood pressure, heart rate  Pulmonary effects: FEV1, FVC  eCO, eNO |
| Ebajemito 2020 ^24^ | 24 DU | UK | RCT PK study 9 day confinement crossover design with 8 PK sessions  Stationary; 1 PK session each day (days 1-8)  Ad lib and controlled use sessions  Inclusion: DU CC/EC; Daily Users of EC/CC, regular CC >1year with no intention to quit  No control arms (CC use included in crossover design) | 9 days | Study EC  Vype ePen3 in 12, 18, 30mg/ml Nic strength  EC ePen 2 as comparator and CC control | Y: Cot (>200ng/mL) at screening | Y: session afternoon before each PK: Allowed to use UB+study product of next PK session and rating of product liking | Subjective liking assessment QA (Product liking)  Product use: liquid used + Satisfaction QA by VAS | Nicotine PK |
| Edmiston 2022 ^25^ | 450 S | USA | RCT parallel-group 12 week controlled switching study with follow up at w 24  Ambulatory, 6 Visits (BL, w1, 3, 6, 9, 12) and 4 follow-up visits (w15, 18, 21, 24)  Ad lib use of EC (exclusive use of test product)  Inclusion: ≥10CPD, CC > 10 years  Control: Continue CC | 3 months + 3m follow-up | Study EC Cigalike EC MarkTen Classic and Menthol  EC vs CC | Y: eCO (8ppm) | Y: 7 days ad lib use prior to study start | None | BoE:  NNAL, TNE, COHb  BoPH: WBC, HDL, 8-i-PGF2a, 11-dh-TXB2, sICAM  FEV1, FVC |
| Eisenberg 2020 ^26^ | 376 S | Canada 17 sites | RCT switching to nic-containing or non-Nic EC for 12 weeks and follow-up to 24 weeks. Switch to study product with counseling. Participants were not required to quit CC immediately at BL. Gradual reduction over treatment period was allowed.  Ambulatory, ad lib use of study EC with 5 clinical visits at BL, week 4, 12, 24, 52 (24 and 52w were follow-ups)  Additional telephone assessments at weeks 1, 2, 8, 18  Inclusion: ≥10CPD, with moderate or strong intention to quit (Motivation to stop scale 5 or higher)  Control: counseling alone (abstinence) | 3 months + 3m follow-up | Study EC NJoy cigalike pen specifically designed for clinical studies  Disposable cartridge  Tobacco flavor 15 or 0 mg/mL Nic  EC vs CC as cessation tool  Counseling alone as comparator | Y: eCO (10ppm) | Y: Only counseling regarding adherence and challenges with use | Point prevalence smoking abstinence: Self-reported abstinence in the past 7d verified with eCO  Continuous abstinence  CPD change from BL  Fagerström Test for Nicotine Dependence (FTND)  Glover-Nilsson Smoking Behavioral QA (to assess behavioral dependence on smoking)  Beck Depression Inventory II (BDI-II; to assess depressive symptoms) | None |
| Fearon 2017 ^27^ | 22 S  18 S | Belfast  UK  LA /  USA | Interventional PK Study 1 in S familiar with EC but no current EC users  Visit 1: Controlled and ad-lib CC  Visit 2: Controlled and ad-lib EC use  Inclusion: ≥10CPD >1year (Study 1)  PK study 2 in experienced EC users who occasionally S  Visit 1: ad lib CC  Inclusion: EC users for at least 3months and occasional S (1-20 CPM)  Control: CC PK session (Study 1/2)  1^st^ gen EC comparator (Study 2) | 3 days | Study EC Vype ePen tobacco flavor  Comparators: 1^st^ gen EC and CC  EC vs CC | Y: eCO (≥10ppm) and Cot (≥200ng/mL) at screening | Y: familiarization for 2days before visit 2 for test product PK | Urge to smoke QA | Nic PK |
| Feng 2022 ^28^ | 11522 adults | USA | PATH Wave 1 (2013/14)  Cross-over evaluation of Nic exposure in tobacco users vs non-users  Population representative cohort | none | No intervention Observational (cross-sectional)  Snus, EC | N: | n/a | None | TNE |
| Ferrari 2015 ^29^ | 20  10 S / 10 NS | Bologna Italy | RCT randomized to CC and EC  Crossover design with 2 use sessions  Inclusion: CC: min. 5 pack-years  Control: CC use session and NS baseline | 2 days | Study EC ELIPS C Series (2^nd^ gen device) with removable cartridge hazelnut flavor without Nic  EC vs CC | N | N | None | FeCO, FeNO  FEV1, FVC, PEF, FEF25, FEF50, FEF75 |
| Flacco 2020 ^30^ | 959 subjects | Italy | Observational, longitudinal study to assess EC long-term efficacy for cessation  CC: ≥ 1CPD ≥ 6 months  EC: ≥ 50 puffs per week ≥ 6 months  Dual Users ≥ 6 months  Data collected by phone/internet | 6 years (2 years follow-up ot previous study) | No intervention Observational (Longitudinal)  EC and DU of CC/EC | Y. eCO | n/a | Quitting rate for any product  Abstinence from CC  CPD  Switching status | Self-reported health QA EuroQoL EQ-D5L (Quality of Life QA)  Occurrence of diseases COPD, angina, myocardial infarction, heart failure, stroke, any cancer (self-reported and/or obtained at visit) |
| Fraser 2015 ^31^ | 1600 S | Australia | 3-arm parallel group RCT  Inclusion: ≥ 5 CPD and willing to try alternative nic products  No control arm with continued CC  Arm A: Quit with medicinal nic  Arm B: Quit or substitute with medicinal nic  Arm C: Quit or substitute with medicinal nic and/or EC  Online survey study; no CRO visit involved | 12 months | Vype EC nic strengths 3.0 or 4.5%  “Vype ECs are unflavored and contain nic, VG, H_2_O and Medicinal Nic | N | N | Continuous abstinence (no CC for > 6 months)  7-day point prevalence abstinence  Use history (medicinal Nic, EC, CC)  CPD  No and length of quit attempts | None |
| Fu 2021 ^32^ | 889 S | Ontario Canada | Cross-sectional study in current S or recent quitters who reported at least one serious attempt to quit smoking by using e-cigarettes in the past 12 months  Online survey; no CRO visit involved | Retrospective 12months | No intervention Observational (Cross-sectional)  EC | N | n/a | Quitting success rate by vaping EC during past 12months  Experiences while vaping to quit smoking with 45 experiences items rated on 7-point scale |  |
| Gale 2019 ^33^ | 180 S | Japan | RCT: Short-term switching study comparing BoE levels before and after switching to HTP  Controlled trial in-clinic. 5 days exposure period **and day 6 PK analysis**  2 day BL of CC followed by randomization to HTP  Inclusion: 10-30 CPD ≥ 3 years no intention to quit  Control: continue CC and abstinence | 6 days | Study HTP  Glo non-menthol and menthol products and IQOS as comparator  HTP vs CC | Y: eCO (>10ppm) and cot (>200ng/mL) at screening | N | Product use: CPD, Sticks per day | BoE:  eCO, Nic+5, NNAL, NNN, 3-HPMA, HMPMA, SPMA, MHBMA, CEMA, HEMA, AAMA, GAMA, 4-ABP, o-tol, 2-AN, 1-OH-Pyr  Nic PK on Day 6 |
| Gale 2022 ^34^ | 295 S + 40 NS | UK 4 sites | RCT ambulatory study at 4 sites. Long-term Switching study for 180 days (longitudinal)  Inclusion: 10-30 CPD ≥ 5 years no intention to quit  Arm A: Continue CC  Arm B: Switch to HTP  Arm D: Cessation  12 visits (BL, and each month)  Sample collection for BoE analysis at BL, months 1, 2, 3, 6 + eCO measures additionally at month 4 and 5  Product use assessed by return of all packs (used or unused) and supply for next month (120% usage of previous period)  Control: CC and abstinence (Arm A and D); cessation with NRT and/or varenicline+counselling | 12 months | Study HTP Glo  HTP vs CC | Y: eCO (≥7ppm) and Cot > 200ng/mL) at screening.  **Study compliance:**  **CeVal**  Reporting of non-compliance in diary encouraged | N | CPD / Stick per day (HTP) | BoE 24h urine & blood  Nic+5, NNAL, NNN, 3-HPMA, HMPMA, SPMA, MHBMA, CEMA, HEMA, 4-ABP, o-tol, 2-AN, 1-OH-Pyr  CeVal / eCO for compliance  BoPH  11-dh-TXB2, 8-i-PGF2a, WBC, sICAM-1, HDL, FeNO, FEV1  NNAL as BoPH defined |
| George 2019 ^35^ | 145 S | UK | RCT randomized to EC with Nic or EC without Nic (longitudinal, ambulatory study)  2 Visits: BL + 1 month  Inclusion: ≥ 15 CPD for ≥ 2 years  Participants with the intention to quit randomized into Nic or non-Nic EC group  Control: CC for subjects no intention to quit | 1 month | Study EC 16mg/mL Nic cartomizer or 0mg Nic cartomizer  EC Vapourlites  EC vs CC | Y: eCO | N | none | FMD  Pulse-wave velocity and augmentation index  Biomarkers by ELISA: Oxidized low-density lipoprotein  CRP, tissue plasminogen activator, platelet activation inhibitor 1 |
| Gmel 2016 ^36^ | 5128 20yr old men | Switzerland | Observational, longitudinal study to investigate efficacy of EC for smoking cessation  Study participants were from 2012!  Data are part of the C-SURF: Cohort Study on Substance Use Risk Factors  Study completely based on QA for BL and 15 month follow-up | 15 months | No intervention Observational (Longitudinal)  EC | N: | n/a | Use behavior  QA EC use at follow-up; CC use at BL and follow-up: CPW; **EC use only characterized by if any or not!?**  Quit attempts  FT-Nic dependence QA | None |
| Goldenson 2021 ^37^ | 17986 S | USA | Longitudinal study with S who recently purchased JUUL EC in 2018 to assess switching rates  Inclusion: Established S by ≥100 cig smoked, smoked in past 30 days, current S some days or every day who purchased JUUL  Online survey: BL and follow-ups at months 1, 2, 3, 6, 9, 12 | 12 months | No intervention Observational (Longitudinal)  EC JUUL | N | n/a | Switching defined as no. CC for ≥30 days  Past 30d Juul use (yes/no)  Risk perception QA (Relative harm of JUUL compared to CC) | No intervention |
| Goniewicz 2017 ^38^ | 20 S | Poland | Interventional switching study  Inclusion: > 5CPD for >12 months with the intention to quit  Visits in-clinic: 3 morning visits at BL, weeks 1 and 2  No control groups | 2 weeks | EC M201 Mild tobacco-flavored cartridges 11.0 mg of Nic, PG/VG 50:50 | N | N | CPD, EC per day (Use consumption)  Minnesota Nicotine Withdrawal Scale (MNWS-R) | BoE Spot urine:  Nic+6 (TNE), NNAL, HEMA, MHBMA, HMPMA, SPMA, 3‑HPMA, AAMA, CEMA, 2‑HPMA, 2-OH-Nap, 1-, 2-, 3-OH-Flu, 1-OH-Phe, 2-OH-Phe, 3-/4-OH-Phe, 1-OH-Pyr  eCO  QA Measures for Health |
| Gorini 2017 ^39^ | 6847 S | Italy | PASSI Survey 2014/2015 in Italian adult population  Inclusion: S who daily or occasionally smoke at time of survey or during preceding 12 months and hat at least 1 quit attempt last 12 months  Interview for quit aid: none, EC, medication (drug or NRT), smoking cessation program  Retrospective evaluation over 12months | 12 months | No intervention Observational (Longitudinal)  EC | N | n/a | Self-reported abstinence for ≥ 6 months  Quit attempts past 12 months | none |
| Guttentag 2022 ^40^ | 84 S | New York USA | RCT for longitudinal analysis of trajectories from CC to EC for cessation efficacy and to analyse use patterns  Subjects assigned to EC with 4.5% Nic or placebo EC without Nic  Advise to reach 50% reduction in CPD within 3 weeks  Fine-grained trajectory approach to examine switching patterns  **SMS services (EMA) used in study for repeated sampling of real-time data. 4 times daily SMS asking about CC and EC consumption for 3 weeks**  4 visits: BL, weeks 1, 2, 3  Inclusion: ≥ 10 CPD, exclusive S interested in using an EC  No control groups | 3 weeks | EC with Nic (4.5 %) and placebo EC 0 Nic  NJOY King Bold  Tobacco-flavor | N | N | EC use per day (self-report)  CPD (self-report)  Heaviness of Smoking Index (HSI) (5-pt score)  Glover-Nilsson smoking behavioral dependence QA  Craving  **CPD used as behavioral indicator to develop trajectory groups gathered via EMA surveys (SMS)**  **Nagin clustering for trajectory analysis** | none |
| Hajek 2019 ^41^ | 886 S | UK | RCT longitudinal trial in adults attending NHS stop-smoking services  S actively seeking help to quit  Randomization into NRT group and EC group (1:1)  Weekly quit support sessions in clinic for at least 4 weeks after quit date  EC group encouraged to experiment with e-liquids of different strength and flavor  Trial visits at BL, week 1, 1m, 6m, 12m  Control: NRT as comparator | 1 year | NRT and EC for cessation  Study EC: Aspire Tank system with Tobacco flavor 18mg/mL Nic  Encouraged to use other strengths and flavors as preferred  NRT: patch, gum, lozenge, nasal spray, inhalator, mouth spray, mouth strip, and microtabs could be selected. Use of combinations encouraged | Y: eCO (≥8ppm) at 1w, 4w, and 1-year follow-up | N: written and oral information how to use EC | Smoking status (self-report)  Use and rating of trial products  Withdrawal symptoms  Primary: 1-year sustained abstinence calculated with Russell Standard as a self-report of no more than 5 CC from 2 weeks after quit date  Secondary: Lower time span of sustained abstinence from 6 to 12m, at 4w, 6m  % Subjects without sustained abstinence from 6m to 12m who reduced CPD by >50%  7-day abstinence  Relapse rate | none |
| Hajek 2022 ^42^ | 1140 S pregnant women | UK | RCT with pregnant women who smoke  2 Arms: EC and Nic Patch (1:1)  Phone calls at weeks 1-4 after intervention and at end of pregnancy  Inclusion: Smoking pregnant women; Daily smokers with intention to quit | End of pregnancy  +  3 months follow-up | NRT and EC for cessation  Study EC: Refillable tobacco flavor EC with 18mg/mL Nic. Further supply for up to 8 weeks.  Optional: 11mg/mL Nic and/or fruit-flavor  Encouraged to use other ECs, strengths and flavors as preferred  NRT: Patch | Y: Cot (saliva) < 10ng/mL at end of pregnancy (primary outcome) for those reporting no nic product use  AB (saliva) < 1ng/mL or eCO <8ppm at end of pregnancy (primary outcome) for those reporting current EC or NRT use | N: Product training was given | Primary: Prolonged abstinence calculated with Russell Standard as a self-report of no more than 5 CC and no smoking at all during previous week at final follow-up  Secondary: Prolonged abstinence at end of pregnancy and 7-day abstinence at 4 weeks and at end of pregnancy  % Subjects without sustained abstinence from 6m to 12m who reduced CPD by >50% | AE in terms of pregnancy and birth |
| Han 2022 ^43^ | 1094 S | USA | PATH Study W1 – W4  1094 S who tried to quit at W1 and were continuing S until W4 | 4 years | No intervention Observational (Longitudinal)  EC | N: | n/a | Quit attempt frequency  Smoking abstinent days | none |
| Harada 2022 ^44^ | 2612 participants (residents) and 722 employees (worksite) | Tsuruoka, Japan | Prospective cohort study (TMCS) in residential and worksite population  Evaluation of HTP use based on QA  Evaluation in 2012-2014 and follow-up in 2018-2019 | 4-6 years | No intervention Observational (Longitudinal)  HTP and DU of CC/HTP | N | N/a | QA based survey: Daily use of CC and HTP  CPD / Sticks per day  Years of smoking / HTP use / Total number of tobacco products used per day  Fagerström test for nic dependence (FTND)  Stage of behavioral change based on QA | FEV1 |
| Hardie 2022 ^45^ | 32 S (23m/9f) | Verona Italy | RCT Nic PK study  4-treatment, 4-period crossover design  4 Treatments and PK visits (one visit per week): 2x glo (different nic strengths); 1x UB CC, 1 Inhaler  Inclusion: S ≥ 10 CPD (non-menthol) ≥1year and UB CC for ≥6 months  Control: UB CC and NRT inhaler as comparator | 1 month | HTP: Glo  Comparators: Nic Inhaler  Control: UB CC  HTP vs CC and NRT | Y: eCO (≥10ppm)  Cot (urine): ≥200ng/mL | Y: 7 day familiarization period after randomization | 5 QA:  Product liking QA (PLQ)  Overall intent to use again QA (OIUA)  Urge to smoke QA (UTS)  Urge for product QA (UFP) (UTS for HTP/NRT)  Product evaluation scale (PES) | Nic PK |
| Harlow 2022 ^46^ | 2229 EC vapers | USA | PATH Wave 2 - 4  Wave 3 was 1 year from BL (W2) and Wave 4 was 2 years from BL (W2)  Longitudinal: Current and former smokers who were EC users at BL (W2)  EC use every day or some days.  Classification of S: Former (> 100 CC lifetime and currently not at all lifetime; Current S: >100 CC lifetime and Every day / some day  All eligible subjects used EC at BL use: Every day / some day | 2 years | No intervention Observational (Longitudinal)  EC | N: | n/a | Use behavior: Smoking / Vaping frequency ->  Use transition odds ratio  Use categories: DU, exclusive use of CC or EC and non-use of both products | none |
| Hatsukami 2020 ^47^ | 264 S | MN, OH, NY  USA | RCT in S no intention to quit  Randomization to either  ad lib EC use with ad lib CC  complete switch to EC  complete switch to NRT (gum) as comparator  6 Visits: BL, weeks 1, 2, 4, 6, 8  continue CC (control)  Inclusion: ≥5 CPD ≥1year  Control: Continued CC  Comparator: Nic Gum (NRT) | 8 weeks | EC Vuse Solo 4.8% Nic  Subjects chose 1 of 4 flavors: tobacco, mint, menthol, berry  Provision of 7 cartridges a week with option to get more in clinic  Comparator NRT  Control CC | Y: eCO (≥10ppm) | N | CPD and product use: report the number of cigarettes smoked the previous day using an Interactive Voice Recording (IVR) system that called participants on a daily basis  FTND  Center for Epidemiological Studies Depression scale (CES-D) | BoE Urine:  TNE  NNAL  Phe-T  CEMA, 3-HPMA, HMPMA, AAMA, 2-HPMA  eCO |
| Hatsukami 2016 ^48^ | 391 S | MN, OR USA | RCT in S interested in switching to snus or nic gum  Randomization to either  Snus or NRT (gum)  8 Visits: BL, weeks 1, 2, 4, 6, 8, 10, 12  BoE measurement at BL and w4  continue CC (control)  Inclusion: ≥10 CPD ≥1year  Control: None  Comparator: Nic Gum (NRT) | 12 weeks | EC  Comparator NRT  Control CC | Y: eCO (≥6ppm) | N | CPD and product use: report the number of cigarettes smoked the previous day using an Interactive Voice Recording (IVR) system that called participants on a daily basis  Minnesota Nicotine Withdrawal Scale, MNWS  7-point Likert-type scale modified from Cigarette Evaluation Scale  Product evaluation scale (PES) | BoE Urine:  TNE  NNAL, NNN |
| Haziza 2020 ^49^ | 160 S | USA | RCT 3-arms parallel group switching study  Controlled study in-clinic for 5 days in confinement and 86 days follow-up ambulatory  Arm A: Menthol HTP  Arm B: Menthol CC  Arm C: Abstinence  BL 1 day UB CC then switch ad lib use  After day 6, discharge and instruction to continue using assigned product or abstinence  During ambulatory part, 3 visits at Days after 1m, 2m, 3m for overnight stay  Inclusion: ?  Control: CC and cessation (Abstinence) | 5 days + 3 month follow-up | HTP: IQOS menthol  HTP vs CC | Y: eCO (10ppm) | N | CPD  FTND | BoPH (Blood, serum, plasma, urine)  WBC  HDL, LDL, TG, TC, ApoA1/B  sICAM  8-i-PGF2a, 11-dh-TXB2  Glucose  HbA1C  Fibrinogen  HCy  Hs-CRP  Blood pressure  FEV1  Weigh, waist circumference  BoE: COHb, 3-HPMA, MHBMA, SPMA, NNAL |
| Haziza 2016a ^50^ | 160 S | Japan | RCT 3-arms parallel group switching study  Controlled study in-clinic for 5 days  Arm A: HTP; Arm B: CC; Arm C: Abstinence  BL 1 day UB CC then switch ad lib use according to study arm for 5 days in-clinic  Inclusion: ≥10 CPD with max 1mg nic yield for at least 4 weeks and CC for ≥3years no intention to quit  Control: CC and cessation (Abstinence) | 5 days | HTP: IQOS  HTP vs CC | Y: eCO (10ppm) | N | CPD / Sticks per day  FTND  modified Cigarette Evaluation Questionnaire (mCEQ)  10-item brief version of the Questionnaire of Smoking Urges (QSU-brief)  Puffing topography by SODIM device | BoE:  COHb, 3-HPMA, MHBMA, SPMA, NNAL, NNN, 3-OH-BaP, 1-OH-Pyr, 4-ABP, 2-AN, 1-AN, o-tol, CEMA, HEMA, HMPMA, SBMA  TNE  CYP1A2 activity |
| Haziza 2016b ^51^ | 160 S | Poland | RCT 3-arms parallel group switching study  Controlled study in-clinic for 5 days  Arm A: HTP; Arm B: CC; Arm C: Abstinence  BL 1 day UB CC then switch ad lib use according to study arm for 5 days in-clinic  Inclusion: ≥10 CPD with max 1mg nic yield for at least 4 weeks and CC for ≥3years no intention to quit  Control: CC and cessation (Abstinence) | 5 days | HTP: IQOS  HTP vs CC | Y: eCO (10ppm) | N | CPD / Sticks per day  FTND  modified Cigarette Evaluation Questionnaire (mCEQ)  10-item brief version of the Questionnaire of Smoking Urges (QSU-brief)  Puffing topography by SODIM device | BoE:  COHb, 3-HPMA, MHBMA, SPMA, NNAL, NNN, 3-OH-BaP, 1-OH-Pyr, 4-ABP, 2-AN, 1-AN, o-tol, CEMA, HEMA, HMPMA, SBMA  TNE  CYP1A2 activity |
| Ikonomidis 2020 ^52^ | 40 S | Athens Greece | RCT 2-arms (1:1) switching to EC vaping or continue smoking  Longitudinal switching study  2 visits: BL and after 4months  Inclusion: Smokers with the intention to quit with average of 25.8CPD  Control: Continue CC | 4 months | EC with 12mg/mL nic  2^nd^ gen EC NOBACCO eGo  EC vs CC | Y: eCO (10ppm) | N | CPD | Platelet function (LTA)  Arterial stiffness  MDA (oxidative stress) |
| Ikonomidis 2018 ^53^ | 70 S | Athens Greece | RCT 2-arms (1:1) switching to EC vaping with and without nic EC  Longitudinal switching study  2 visits: BL and after 1month  Inclusion: Smokers with the intention to quit with average of 24CPD  Control: 20 smokers of CC | 1 month | EC with 12mg/mL nic  2^nd^ gen EC NOBACCO eGo  EC vs CC | Y: eCO (10ppm) | N | CPD | Heart rate  Aortic stiffness (Pulse wave velocity; PWV and augmentation index)  MDA (oxidative stress)  eCO |
| Jankowski (2022) ^54^ | 1090 individuals (representative population) | Poland | Cross-sectional study nationwide survey on tobacco use: Global Adult Tobacco Survey (GATS); a QA with 12 questions on tobacco, HTPs and ECs  Tobacco use prevalence survey  Smoking status divided into  current smokers: daily/occasional  former smokers  Non-smokers  Current (daily) users of EC, HTP | n/a | No intervention  Observational (Cross-sectional)  EC / HTP | N | n/a | Smoking patterns: type of product used; CPD correlated with socio-economic data to associate prevalence and ORs  EC / HTP prevalence: daily use of the product | none |
| Jay 2019 ^55^ | 90 S | Nebraska  USA | RCT, open-label, parallel-cohort, switching study under confinement  Subjects randomized to 6 groups (15 each):  Ad lib use of EC in 4 different flavors (one per group)  Confinement for 5 days (exposure assessment) and additional day for PK assessment  Exposure BL vs Day 5  PK on Day 6  Inclusion: ≥10 CPD for ≥1year  Controls: Continue CC and abstinence | 6 days | EC JUUL with 50 mg/mL Nic  In 4 different flavors (tobacco, mint, mango, crème)  EC vs CC | Y: urine cot (≥500ng/mL)  eCO (>12ppm) | N: But 5 days use before PK | FTND  Wisconsin Inventory of Smoking Dependence Motives (WISDM)  Severity of urge to smoke by 100-point visual analog scale (VAS) | BoE (urine)  TNE  NNAL, NNN  3-HPMA, MHBMA, SPMA, HMPMA, CEMA  1-OH-Pyr  COHb  Nicotine PK |
| Kaplan 2021 ^56^ | 6794 S | USA | Longitudinal analysis of PATH data Study Wave 3 and 4  Smokers of CC at W3 who use EC or NRT to quit between W3 and W4  Cessation definition: Smoker at W3 and former smoker at W4 | 1-2 years | No intervention Observational (Longitudinal)  EC / NRT | N | n/a | Quit rate  Motivation to quit QA  Wisconsin Inventory of Smoking Dependence Motives (WISDM)  Smoking prevalence in W4 and ORs | none |
| Kimber 2021 ^57^ | 50 S | London UK | RCT Switching study to 1 of 3 ECs:  Cigalike 18mg/mL Nic  Tank 18mg/mL Nic  Tank 6mg/mL Nic  Ambulatory study with 3 Visits: BL, weeks 1 and 2  Inclusion: ≥5CPD for ≥1year with the intention to quit  Controls: None | 2 weeks | ECs with tobacco flavor: Cigalike 18 mg/mL Nic and tank system EC with 18mg/mL or 6mg/mL Nic strength | N | Y: only instructions how to use EC | Puffing topography captured via videorecording: Number of puffs, puff duration, inter-puff interval  Motivation to stop Scale (MTSS)  FTND  CPD  Craving and withdrawal symptoms: Mood and Physical Symptoms Scale (MPSS)  2-part VAS | eCO |
| Kotz 2022^58^ | 2740 S and ex-S (quitters) | Germany | Longitudinal survey: German Study on Tobacco Use (DEBRA)  Representative Population  Inclusion: Current S and recent quitters (<12months since quitting) who made ≥1 attempt to quit in past 12months | 12 months observed | No intervention Observational (Longitudinal)  EC / NRT | N | n/a | Prevalence of cessation aid in quitters:  Counseling, EC with or without nic, NRT, drugs  ORs  Self-reported smoking status | None |
| Krautter 2015 ^59^ | 167 S | 3 sites FL, IN, WI  USA | RCT Switching study under confinement  Ad lib use of assigned product  Randomization to 1 of 5 use groups:  Dual Use with max of 40% CPD of BL (Snus+CC)  Exclusive use groups of each individual smokeless test product  Exposure Assessments: BL vs Day 5  Use behavior assessments each day (BL+ D1-5)  Inclusion: ≥10 CPD for ≥1 year  Control: Abstinence | 6 days | Smokeless Camel Snus  Sticks, Strips, Orbs  Smokeless vs CC | Y: Cot (urine): ≥200ng/mL | Y: Only short testing for palatability | CPD in DU group  Product use: Mouth-level exposure (MLE) for CC  Units consumed  Self-reported Minnesota Nicotine Withdrawal Scales-Revised (MNWS-R) | Urine mutagenicity (Ames)  BoE (Urine and faeces)  TNE  TSNAs (NNAL, NNN, NAB, NAT)  AA (3-ABP, 4-ABP, 2-AN, o‑tol)  OH-PAHs (1-OH-Pyr, 1-/2-OH-Nap, 1-/2-/3-/4-/9-OH-Phe, 2-OH-Flu  AAMA, GAMA, 3-HPMA, SPMA, MHBMA, DHBMA, HEMA, CEMA  Plasma Nic/Cot |
| Krishnan 2022 ^60^ | 1536 EC users | USA | Longitudinal analysis of PATH data Study Wave 3 and 4 in established EC users at W3  EC and CC use evaluated at W4 for EC users of W3  Cigarette smoking status at W3:  NS, Former smoker, current smoker  Current S: ≥1CC in past 30days  Former S: No CC past 30days but > 100 CC lifetime  NS: No CC past 30days and < 100 CC lifetime  EC current use: Every day or someday | 1-2 years | No intervention Observational (Longitudinal)  EC | N | n/a | Cigarette smoking status and Dual product use at W4 for EC users  Daily EC use prevalence in NS, Former S, current S  W4 Smoking and EC abstinence at W3 EC users | none |
| Kumral 2016 ^61^ | 98 S | Turkey | RCT Longitudinal trial; Switching study  Randomization to 2 groups EC and abstinence | 3 months | EC vs abstinence | N | N | none | Sinonasal symptom scores  Mucociliary clearance (MCC) by saccharin-transit time (STT) |
| Lechner 2015 ^62^ | 22 S | OK  USA | RCT Cross-over design in exclusive S with no experience in EC vaping  Switching study cross-over using a 1^st^ gen EC and a 2^nd^ gen EC on 2 separate days (one session per product)  Session: 5min ad lib use of EC  Two sessions separated by 48hours  Inclusion: ≥5CPD and no intention to quit; no controls | 2 days | Study ECs:  1^st^ gen EC: Blu tobacco flavor 16mg/mL Nic  2^nd^ gen EC: Tank system JoyeTech E-go tobacco flavor 16mg/mL Nic | Y: eCO (10ppm) | N | Smoking history QA: No. of quit attempts, CPD  FTND  Craving and Nic withdrawal: Mood and Physical Symptoms Scale (MPSS)  Confidence to quit smoking scale | None |
| Lee 2019 ^63^ | 150 S | South Korea | RCT Longitudinal switching study in Smokers with the intention to quit  Recruitment from 1 company  Smokers assigned to 1 of 2 groups:  EC use or Nic Gum (NRT) 1:1  Visits every 4 weeks for evaluation and counseling  3 Visits for QA assessments: BL, months 3 and 6  Inclusion: ≥10 CPD for ≥1year and S for ≥3years with intention to quit  Control: NRT (nic gum) | 6 months | EC: eGO-C 0.01mg/mL Nic  Comparator: Nic gum  EC vs NRT | Y: eCO (10ppm) and urine Cot at BL and 6months | Y: 50 min education / Training session | Continuous abstinence at 9-12 weeks and 9‑24 weeks  7-day point prevalence of abstinence at weeks 12 and 24  Reductions in CPD | None |
| Li 2020 ^64^ | 886 S | England UK | RCT Longitudinal study  Randomization to EC or NRT group (comparator)  QA assessments at BL, months 6 and 12  Visits every week for treatment delivery and support sessions  Inclusion: Smokers with the intention to quit | 12 months + 12 months follow-up | EC One Kit device 18mg/mL Nic 30mL bottle  Further supply purchased on their own  NRT as comparator  EC vs NRT | Y: eCO at 12months | Y: 1hour training session | Quality-adjusted life years (QALYs): 5-level EuroQol 5-dimension QA (EQ-5D-5L)  EQ VAS scale  eCO validated abstinence rate at 12months | Quality-adjusted life years (QALYs): 5-level EuroQol 5-dimension QA (EQ-5D-5L) |
| Liu 2021 ^65^ | ? | Maryland USA | RCT Single-center, randomized, controlled, switching study in-clinic under confinement  Switch from CC to ad lib use of EC  3 arms: Non-menthol EC, menthol EC, Nic gum  Daily use assessments  Metabolomics assessments BL vs Day5  Inclusion: ≥10CPD with 1^st^ CC within 30min of waking  Control: Nicotine gum (NRT comparator) | 7 days | EC: Vuse 1^st^ gen cigalike tobacco or menthol flavor  4.8% Nic  EC vs CC | N | N | None | Metabolomics in plasma and urine |
| Lucchiari 2020 ^66^ | 210 S | Milan Italy | RCT recruited from COSMOS II program (Continuous Observation of Smoking Subjects)  3 groups: EC, placebo CC (no Nic), only support  EC group ad lib EC during w1 and no CC use for w2-12  Non-Nic CC placebo double-blinded  Support only group as control  Phone-counseling (support) at weeks 1, 4, 8, 12  Visits at clinic: BL and 3 months  Follow-up at months 6 and 12 to assess smoking behavior and pulmonary health  Inclusion: 55years or older, ≥10CPD ≥10years  Control: Support only (abstinence) | 3 months + 9 months follow-up (overall 12m) | EC 8mg/mL Nic | Y: eCO (7ppm) | N | Continuous abstinence (verified with eCO)  FTND  4-item motivational QA (motivation to quit smoking)  Hospital Anxiety and Depression Scale (HADS)  EC use QA | Leicester Cough QA  Respiratory Symptoms |
| Lüdicke 2019 ^67^ | 984 S | 20 sites USA | RCT ambulatory trial 2-arms in healthy S no intention to quit  Switching study to HTP  Randomization to 1 of 2 groups:  HTP or Continue CC  Further stratification to DU group  DU: 1-70% HTP use  Switchers to HTP instructed to use HTP exclusively  Visits for monthly safety checks  Assessments at 3 visits: BL, months 3 and 6  Inclusion: ≥10CPD for ≥1year and smokers for ≥10years  Control: Continue CC | 6 months | HTP IQOS  HTP vs CC | Y: Cot (urine) ≥200ng/mL at screening | Y: Training on use of HTP and ad lib use for 8days before start | Electronic use diary: CPD / Sticks per day  Prevalence of use after 6 months for HTP arm | BoPH:  HDL-C, WBC, sICAM-1, COHb, FEV1  11-dh-TXB2, 8-i-PGF2a  BoE:  NNAL, NNN, eCO, MHBMA, HMPMA, CEMA, 3-HPMA, 3-OH-BaP, 1-OH-Pyr  TNE |
| Lüdicke 2017 ^68^ | 40 S | Poland | RCT Switching study under confinement  Randomization to HTP or continue CC (1:1)  Ad lib product use  Exposure assessment at BL and Day 5  Use behavior (BL, Day 4)  Use/Consumption: each day  Inclusion: ≥10 CPD for ≥4weeks  Control: Continue CC | 5 days | HTP IQOS  HTP vs CC | N | Y: Trial of study product prior to randomization | Daily product use: CPD / Sticks per day  Product use behavior (SODIM device): Puff duration, interpuff interval, puff volume, number of puffs, total volume  Modified Cigarette Evaluation QA (mCEQ) | BoE (urine)  NNAL, NNN, 3-HPMA, CEMA, SPMA, MHBMA, 4-ABP, 2-AN, o-tol, 1-OH-Pyr  TNE  Blood:  COHb  Nic, Cot, OH-Cot in Plasma  Caffeine, paraxanthine plasma |
| Luk 2021 ^69^ | 1213 S | Hong Kong | Longitudinal observational study  Randomization in terms of counseling but no product intervention  HTP user classification: Use on at least 1 of last 7 days; HTP initiation: Never user at BL and use in past 30 or 7 days at follow-ups  Visits: BL visit and telephone follow-ups at months 1, 2, 3, 6  Inclusion: ≥1CPD for ≥3months; S with the intention to quit or reduce CC  Control: None | 6 months | No intervention Observational (Longitudinal)  HTP  Intervention only by means of quitline service | Y: eCO (≥4ppm) at BL | N | QA for CC use and dependence: Heaviness of Smoking Index at BL  7-day point prevalence abstinence at 6months  Quit attempts  HTP use pattern: On how many days in last week; Use in past 30 / 7 days | None |
| Manzoli 2017 ^70^ | 1355 participants | Italy | Prospective cohort study  Longitudinal trial with S, EC users and DU  Data collection through QA by phone interviews or via internet  CC at BL and after 24 months were observed  Inclusion: CC: ≥1CPD for ≥6months  EC: EC use for ≥6months  DU: DU for ≥6months | 24 months | No intervention Observational (Longitudinal)  EC | Y: eCO in random sample of abstinent subjects at 12 and 24months | n/a | Sustained abstinence of 30 days from CC at 24months  Abstinence from both CC and EC  CPD  Proportion of participants reducing CPD by >50% between BL and 24m  Self-reported health: EuroQol EQ-D5L  Switching rate  Quit rate | Self-reported health: EuroQol EQ-D5L |
| Martner 2019 ^71^ | 12 S | FL  USA | Contingency Management (CM) Interventional Trial  Nonconcurrent multiple baseline across participants design  3 Phases: BL, EC, EC+CM  BL: random BL of 2-8 days for training of breath sample collection for CO and familiarize with study procedures  EC: Quit smoking and use EC as needed for 14 days  EC+CM: 14 days of EC use  Online survey every 4 days  Daily measurement via app  Inclusion: ≥8 CPD for ≥2years with the intention to quit | 30 days | EC Smokio or Joeyetech eMode 24mg/mL Nic  Tobacco flavor or menthol flavor  EC + CM (Contingency Management) | Y: eCO (4ppm) for abstinence  Daily self-applied sampling video-verified via app | Y: Instructions of use | Bluetooth-enabled EC for puff monitoring by web app Motiv8  Puffs per day  eCO verified abstinence by video sample collection of breath analysis using Motiv8  Acceptability ratings: 23-item QA about participants’ experience with the EC (ease of use, helping to quit, reductions in cravings etc)  Behavior change inventory: Online survey about withdrawal, AE, medication, use of other tobacco products, new methods to initiate or maintain abstinence? | eCO |
| McEwan 2022 ^72^ | 35 DU of snus and CC | Sweden | RCT Crossover design for Nicotine PK under confinement  1 product use session each day:  NP: 60min use of product; for CC: 1 CC ad lib for 5min  Inclusion: Daily snus users (pouch weight ≥ 0.8 g, containing ≥ 8 mg nicotine) under their upper lip for at least 6 months + S ≥5 CP**W** S for ≥1year  Comparator: 4 NPs  Control: CC | 8 days | Nicotine pouch Lyft mint 10mgNic/pouch  4 comparator Nic pouches from SM, JTI, Imperial, Altria  Nicotine pouch vs CC | N | Y: NP were given on admission to clinic  Period of 30 min for familiarization with each test product | Subjective effects: Product liking QA | Nic PK |
| McRobbie 2015 ^73^ | 40 S | London UK | Interventional switching study to EC in S with the intention to quit CC; Instructed to use EC ad lib for quitting  6 Visits: BL 1w prior to target quit date; Target quit date (week 0), weeks 1, 2, 3, 4  Each visit: QA (MPSS), use behavior and eCO  BL and w4: Exposure assessment (BoE)  **10 subjects PK after 1^st^ use and week 4**  Inclusion: S who were with the intention to quit and not using EC | 4 weeks | EC Green Smoke 2.4% nic, 1^st^ gen cigalike | Y: eCO (10ppm) | Y: instructions to use | Mood and Physical Symptoms Scale (MPSS): for tobacco withdrawal symptoms and urge to smoke  CC use and EC use (cartridges/d; Mean number of days EC used over previous week) | BoE (urine)  3-HPMA  eCO  Cotinine  Nic PK |
| Meier 2017 ^74^ | 24 S | USA | Double-blind RCT Crossover design  Ambulatory setting  CC use as usual for 1 week, followed by 2 weeks ad lib use of placebo EC (1week) and 1^st^ gen EC device (1 week)  Instructed to use EC if they want; smoke regular CC if you wish  Weekly visits at the clinic; total of 4 visits: BL, weeks 1, 2, 3  QAs, BoE and eCO at each visit  Inclusion: ≥10CPD no intention to quit, no EC users but interested in using EC  Control: Placebo EC (non-Nic) | 3 weeks | EC BluCig 16mg Nic or 0mg Nic (placebo)  Tobacco or menthol flavor depending on UB CC | Y: eCO (≥6ppm) at screening | N | Use diaries of EC and CC use: “real time” assessment by filling daily the diary: No of EC puffs per use/episode; no. of episodes; total puffs/d; % of participants using EC 6+ days/week  CPD and quit attempts: Timeline Follow-Back QA at each visit  Heaviness of Smoking Index (HSI) was calculated using cigarettes per day (CPD) + time to first cigarette  Smoking resistance task: demonstrate 12-h abstinence from all tobacco  Nine-item Minnesota Nicotine Withdrawal Scale for craving and withdrawal symptoms  37-item Brief Wisconsin Inventory of Smoking Dependence Motives (B-WISDM) to assess level of tobacco dependence at baseline  Glover-Nilsson Smoking Behavioral QA  modified Cigarette Evaluation Scale (mCES) for liking and acute effects  Intention to quit | eCO  Cot (urine) |
| Meltzer 2017 ^75^ | 2900 DU of EC/CC | USA | RCT with three arms of different quitting strategies. 1 arm includes information about ECs.  Cessation trial longitudinal  Mail and E-Mail based assessment: BL + follow-up assessments at 3-month intervals (months 3, 6, 9, 12, 15, 18, 21, 24)  Inclusion: DU of CC/EC: CC ≥1CP**W** ≥1yr and EC use ≥1/week over past month | 24 months | Cessation guides including EC use brochures | Y: eCO (8ppm) and Cot (saliva): 10ng/mL at 12m and 24m to verify abstinence | N | Use history of EC and CC use  QA / Assessments:  FTND  Intention to quit  Motivation to quit tobacco cigarettes is assessed using the Stages of Change Algorithm, and a continuous measure of readiness to quit, the Contemplation Ladder  situation-specific abstinence self-efficacy scale; a measure of abstinence-related motivational engagement (ARME); and a measure of tobacco cigarette expectancies | none |
| Miura 2015 ^76^ | 60 S / 20 NS | Japan | RCT Switching study confinement  Switch to Tob Inhaler/Continue CC (2:1)  Ad lib use throughout the day  6 Assessments: BL, days 1, 8, 15, 22, 29  Inclusion: ≥20CPD ≥1year of 1mg tar CC  Control: NS and CC | 1 month | Tobacco Inhaler VS CC | Y: Cot (serum) 14ng/mL at screening | N | CPD  Puffs/day | BoE (plasma):  SCN, Cot  COHb  BoE (Urine):  TNE  NNAL, 3-HPMA, HMPMA, MHBMA, DHBMA, SPMA, TMA, 1-OH-Pyr, 4-ABP  Mutagenicity (Ames test) |
| Morphett 2022 ^77^ | 1563 S | Australia | RCT Cessation Study with 3 arms  Arms A/B: Cessation or quit advise with NRT  Arm C: Quit advice with NRT + EC  Inclusion: ≥6CPD | 7 months + 5 months follow-up (overall 12m) | EC Disposable EC 3.0% or 4.5% Nic strength  Unflavoured | ? | ? | Continuous abstinence  Self-reported 7-day point prevalence abstinence  NRT and EC use  Motivation to quit smoking  CPD  Product use  Quit attempts | None |
| Morris 2022 ^78^ | 79 S | NE, NJ USA | RCT Crossover design under confinement  Exposure assessment at BL, days 9 and 14  9 days of exclusive ad lib EC use and  Exclusive EC use / DU / CC for day9-14  Inclusion: ≥10CPD for ≥1year no intention to quit  Control: Continue CC arm | 14 days | EC Myblu pod device in 5 Nic strengths: 12, 24, 25, 36, 40 mg/mL with different flavors  EC vs CC | Y: Cot (urine): 200ng/mL  eCO (10ppm) at screening | Y: 30 min training session with device | Use documentation daily | BoE:  COHb  NNAL, 3-HPMA, SPMA, CEMA, HEMA, HMPMA, MHBMA, o-tol, 1-AN, 2-AN, NNN, 1-OH-Pyr, 3-OH-BaP  TNE |
| Myers Smith 2022 ^79^ | 135 S | London UK | RCT Switching study Longitudinal trial  Randomization 1:1 EC/NRT  Visits at site: BL and week 1  Bring EC at week 1 for advice on use, test and start actual product use at w1  **Set target quit date at week1**  Follow-up by phone call: months 1, 6  Inclusion: S with the intention to quit with history of failed quit attempts  Control: NRT arm | 6 months | EC Refillable starter pack and voucher to purchase EC of their choice. Flavor / strength as they chose. Encouraged to try other EC if dislike of 1^st^ purchase | Y: eCO at week4 and 6months for subjects reporting over 50% CC reduction | Y: After BL instruction on use of purchased EC at site at week1 | CPD: self-report in the follow-up  at 1, 4 and 24 weeks  Primary outcome: CPD reduction of at least 50% at 6months  Abstinence from smoking t 4weeks  Sustained abstinence at 6months (no more than 5CC smoked since week 4)  EC / NRT use  Smoking behavior  % participants continuing product use at 6months  Liking (product rating)  How helpful is product to quit rating | Respiratory symptoms checklist |
| Nides 2014 ^80^ | 29 S | USA | Interventional study  1 week ad lib use of EC after 1 week of CC use BL measurement  3 Visits at clinic: BL, week 1, week 2  Inclusion: ≥10CPD ≥1year with no intention to quit  Control: None | 2 weeks | EC Cigalike  1^st^ gen type NJOY King Bold 52mg/mL Nic  Menthol or non-menthol | Y: eCO (10ppm) at screening and visit 3 (w2) | Y: Initial training at week1 with EC for 20 min. 1 week ad lib use before Nic PK assessment | CPD and puff/d self-reported in use diary  EC perception QA: 9-tem QA  Craving assessment: QA of smoking urges (QSU)  Withdrawal assessment: Minnesota Nicotine Withdrawal Scale (MNWS) | Heart rate  Nic PK at visit 3 |
| O’Connell 2016 ^81^ | 105 S | USA | RCT Switching study under confinement switch to ad lib EC use or DU  Randomization to either 1 of 3 exclusive EC product use groups or DU groups  DU groups required reduction of min 50% in CPD  Inclusion: ≥10 CPD for ≥1year  Control: Abstinence (cessation) | 5 days | EC blu EC tobacco, menthol and cherry flavor  24mg/mL Nic  and DU | Y: eCO (12ppm) and Cot (urine): 500ng/mL at screening | Y: Training | CPD and EC use  Urge to smoke as measured by VAS  Modified Cigarette Evaluation Scale | BoE:  COHb  NNAL, 3-HPMA, SPMA, CEMA, HMPMA, MHBMA, NNN, 1-OH-Pyr  TNE  Nic, Cot, OH-Cot (Plasma)  eCO, eNO  FVC/FEV1  Blood pressure, heart rate |
| O’Connell 2019 ^82^ | 15 S | USA | RCT Crossover study for Nic PK evaluation under confinement  D1: UB CC  Controlled EC use on Days 2-6 for product PK  Inclusion: ≥10 CPD for ≥1year with no intention to quit  Control: CC (UB) | 6 days | EC 5 types of blu EC in different nic strengths: 16, 25, 26, 40, 48 mg Nic  4 podsystems (myblu) and 1 open system (blu PRO) | Y: eCO (12ppm) and Cot (urine): 500ng/mL at screening | N | Subjective effects QA | Nic PK |
| O’Connor 2018 ^83^ | 151 S | USA | RCT study  Allowed to smoke during product use first week  Return to clinic 1 week later. Subjects with use of 1+ unit of snus per day eligible for study  Randomization after the 1^st^ week to 1 of 5 arms:  Arms based on intended substitution of CC (full / partial); use (specific / ad lib); and continued CC  3 visits: BL, week1 (familiarization week), week2 (randomized trial)  Inclusion: ≥10 CPD and no smokeless tobacco for 3months  Control: Continue CC  **Conclusion: Short-term testing does not predict continued or long-term use** | 2 weeks | Camel Snus menthol and non-menthol | N | Y: trial phase of 5 min at BL to chose product based on preference  Test product for 1week for eligibility to continue in study | Product use by interactive voice recording  CPD  Product Evaluation Scale (PES)  Drug Effects Questionnaire (DEQ) | none |
| Odani 2023 ^84^ | 7044 participants | Japan | Longitudinal, nationwide internet-based survey  Observational study over three waves  Classification: CC: past 30 day S  Former S / Never S  Assessment of ≥1 month and ≥6months quitting/smoking cessation and smoking relapse at 1 year follow-up in relation to HTP use at BL | 2 years | No intervention Observational (Longitudinal)  HTP | ? | n/a | CPD / Sticks per day  Association of HTP use with cessation and relapse of CC  APR and AOR assessed | none |
| Ogden 2015 ^85^ | 163 S | USA | RCT Switching Study to HTP or Snus  13 Visits: BL, weeks 2, 4, 6, 8, 10, 12, 14, 16, 18, 20, 22, 24  24h confinement at BL, months 3 and 6 (exposure assessment BoE sample collection)  Inclusion: ≥15CPD for ≥10years no intention to quit  Control: CC (ultra-low machine yield tobacco burning CC) and NS | 6 months | HTP Eclipse regular and menthol  Snus: Camel Snus Frost, Spice and Original  CC: Camel Ultra Lights (regular); Salem Ultra Lights (menthol) | Y: Cot (urine): 50ng/mL to verify NS at screening  eCO every 2 weeks  No compliance during study (only self-report) | Y: Orientation information  Choice of favored product in each category at BL and supplied every 2 weeks at visits | Product use diary (daily tracking; review by clinic staff every 2 weeks)  Product use compliance rate (self-report) | Health status QAs:  St. Georges Respiratory Questionnaire (SGRQ)  Leicester Cough Questionnaire (LCQ)  BoE: TNE, COHb, eCO  Spirometry  Lipid/cardiac risk markers, hypercoagulable state markers, endothelial function, DNA damage, and carboxyhemoglobin  Smoking Cessation Quality of Life Questionnaire (SCQoL) |
| Ohmomo 2022 ^86^ | 208 participants  52 per group of HTP / NS / CS / Former S | Japan | Longitudinal cohort study in 11000 individuals BL survey 2012-2015  Blood collection in 2084 participants at follow-up 2018/2019  Exclusive HTP users in follow: 52 switchers from CC to HTP  These were matched (gender, age, drinking habits, propensity score) with 52 NS / S / Former S at follow-up  Former S match with HTP in number of years since quitting; NS match with BMI, blood pressure and other health markers; S with product use | Ave. 1.7 years | No intervention Observational (Longitudinal)  HTP; specifically glo, IQOS, ploom | Y: Cot (urine) to verify NS and correlation with methylation sites | n/a | None | DNA methylation markers  Transcriptomics / gene expression alterations |
| Oncken 2015 ^87^ | 27 S | USA | RCT Crossover switching study to EC in S no intention to quit  1 week use of 1 EC followed by crossover to 2^nd^ EC for another week  Subjects shall abstain from CC  3 Visits: BL, weeks 1 and 2  Inclusion: ≥10 CPD  Control: None | 2 weeks | EC Joye Ego C 18mg/mL Nic tobacco or menthol flavor | Y: Cot (plasma) at BL | Y: Instruction to use  Nic PK performed after 1 week of use for each EC | Use diary (self-report): puffs/h; CPD  QA during PK assessment:  VAS for: momentary perceptions of desire to smoke, restlessness, irritability, trouble concentrating, and feeling calm or relieved, recorded on visual analogue scales  Cigarette Impressions QA (Likert-scaled QA) for liking of product | Pulmonary function: Body phlethysmography  eCO, heart rate, blood pressure  Nic PK |
| Osibogun 2020 ^88^ | 1870 participants | USA | PATH Wave 1 (2013/14) and Wave 2 (2015/16)  Longitudinal: DU at W1 and trajectories at W3 to no use of any tobacco/nic product (cessation transition) / exclusive EC (harm reduction transition) / exclusive CC (cigarette transition)  Classification of DU: Past 30 days use of EC and CC  Transitions: No or exclusive use of one product over past month | 2 years | No intervention Observational (Longitudinal)  EC | N: | n/a | Use behavior: transition in smoking behavior of dual users by multivariate prediction based on demographic and behavioral factors like age, sex, race, education etc and first exposure to tobacco, duration of use, marijuana and alcohol consumption  QAs:  Nicotine dependence symptoms QA  Interest in quitting (motivation to quit)  History of CVD illnesses | none |
| Pacifici 2015 ^89^ | 34 S | Torino Italy | Interventional switching study in S with the intention to quit CC  Uncontrolled design / Interventional  Participants were supplied with EC at BL and given proper training.  Subjects encouraged to use EC but allowed to smoke CC as well  Counselling in week 1  Own EC use in week 2 and 3 to substitute for CC  Week 4: Complete substitution of CC by own EC use  Whatsapp chat group with all participants and study staff  Stratification into exclusive users of either EC or CC and DU  **DU not further stratified**  4 Visits: BL, months 1, 4, 8  Inclusion: ≥10 CPD for ≥ 4 years  Control: CC remainers; not included as control but kept in study although no use of EC | 8 months | EC AVATAR with cartomizer and PUFFIT liquid in varying nic contents (according to subjects CPD)  Flavor chosen by participants  Interventional: Type of EC and nic strength may change over time | Y: eCO  Plasma Cot and OH-Cot at baseline and month 1, 4, 8 | Y: Use instructions at BL and training. Specific training in first week on use of nic-free EC | FTND  CPD at BL vs TP month 1, 4, 8  Cessation rate | Plasma Cot, OH-Cot |
| Pierce 2020 ^90^ | 2443 S | USA | PATH study evaluation of S at W1 who reported quit attempt before W2 and completed W3  Assessment of abstinence at W3  Ever and current use assessed at W1, W2, W3 for CC and EEC and other tobacco (smokeless, cigars etc.)  Current use: every day, somedays, not at all | 2 years | No intervention Observational (Longitudinal)  EC | N | n/a | 12+ months abstinence from CC and EC and all forms of tobacco  30+ days abstinence from CC and EC and all forms of tobacco  QA on methods used to quit | none |
| Polosa 2015 ^91^ | 71 S | Italy | Prospective switching study (interventional) in smokers buying EC for 1^st^ time  Subjects were encouraged to use EC to reduce CPD  **Characteristics of EC type and liquid (flavor, nic) were well characterized in this study**  3 Visits: BL, months 6, 12  Inclusion: Criteria not reported  No controls | 12 months | EC free choice of nic strength and flavor | N | Y: Instructions on use | Sustained 50% reduction on CPD: reducers; sustained 80% reduction in CPD: heavy reducers  And quitters  Self-reported reduction all compared to BL | none |
| Polosa 2014a ^92^ | 50 S  38 eligible at 6 months | Italy | Prospective study (interventional) smokers switching to EC with no intention to quit CC. Subjects encouraged to use EC but allowed to smoke CC as well  Provision of study EC with liquid for 1 month. Max ad lib use of 5mL liquid per day.  CPD shall be reduced by EC use  Further refill of e-liquid at visits  5 visits: BL, months 1, 2, 3, 6  Inclusion: ≥15 CPD for ≥10 years with no intention to quit | 6 months | EC 2^nd^ gen EC tobacco flavor 9mg/ml nic | Y: eCO (10ppm) to verify self-reported abstinence | Y: Instructions on use | Sustained 50% and 80% reduction in CPD at week-24  Abstinence rate  Self-reported in diary  QA rating of usefulness / liking of EC by 10-pt VAS | none |
| Polosa 2014b ^93^ | 40 S  23 eligible at 6 months | Italy | Prospective study (interventional) smokers switching to EC with no intention to quit CC. Subjects encouraged to use EC but allowed to smoke CC as well  Provision of study EC with liquid for 1 month. Max ad lib use of 4 cartridges per day.  CPD shall be reduced by EC use  Further refill of e-liquid at visits  5 visits: BL, months 1, 2, 3, 6  Follow-up visits at 18 and 24 months to record use behavior (EC and CPD)  Inclusion: ≥15 CPD for ≥5 years with no intention to quit  No controls | 6 months + follow up at 18 and 24 months | EC 2^nd^ gen EC Categoria tobacco flavor 7.2mg/ml nic | Y: eCO (10ppm) to verify self-reported abstinence  And 15ppm at screening | Y: Instructions on use | Sustained 50% and 80% reduction in CPD at week-24  Abstinence rate  Self-reported in diary  QA rating of usefulness / liking of EC by 10-pt VAS | none |
| Price 2022 ^94^ | 871 S  **Lost to follow up: 794 at 12m!!!** | UK | Mixed-methods evaluation  Interventional study using ECs as cessation aid given to S free-of-charge in pharmacies  Free equipment and refill for ad lib use to quit CC  **Subjects recruited through pharmacies who have experience in delivering smoking cessation services**  3 Visits: BL, weeks 2, 4  Follow-ups at 3 and 12 months  No controls | 4 weeks + follow-ups at 3 months + 12 months | EC | Y: eCO verified status at BL and 4w | Y: Support in counselling and EC use by pharmacy | Smoking abstinence  Smoking reduction (CPD)  Quit rates  By interviews / QA  Self-reported abstinence eCO verified | none |
| Prokopowicz 2020 ^95^ | 90 volunteers | Poland | Cross-sectional study (uncontrolled)  Stratification into:  CC for ≥2years  DU CC for ≥2years and EC for ≥6months  EC: EC for ≥6months and former CC (Switchers from CC to EC)  NS  Control: NS | 1 visit | EC and DU | Y: eCO | n/a | QA based:  CPD  Puffs/day  Puffs/session  Liquid/week | 11 metals in urine:  Cr, V, Ba, In, Ag, Co, Ni, Mn, Pb, Sb, Cd |
| Pulvers 2020 ^96^ | 186 S | USA | RCT comparing 6w of EC vs CC use  Switching study (interventional)  Counseling (action planning) to switch completely to EC  Provision of pods at BL and week 2  Randomization to EC/CC (2:1)  3 Visits: BL, weeks 2 and 6 + phone calls at weeks 1 and 4  **DU were defined as any EC + CC use last 7days**  Inclusion: ≥5CPD on 25 of past 30d for ≥6months interested into switching  Control: Continue CC | 6 weeks  +  6 months follow-up | EC JUUL 4^th^ gen EC with nic salt pod system  5% nic in different flavors could be chosen  EC vs CC | Y: eCO (6ppm) | Y: education and training | Switching rates at weeks 2 and 6  Past 7-day CPD  Exclusive EC use was defined as no CC past 7 days  Follow-up phone call to assess switching rate without biochemical verification | BoE:  NNAL  Cot  BoPH:  Respiratory symptoms (American Thoracic Society Questionnaire), lung function (FEF), blood pressure |
| Ramstrom 2016 ^97^ | 28302 subjects | Sweden | Long-term observational study in Swedish general population by QA  5 categories of CC and snus users:  Daily CC, no daily snus  Primary daily CC, secondary daily snus  Daily snus, no daily CC  Primary daily snus, secondary daily CC  No daily tobacco use  **Data regarding decade of birth used to establish cohort-specific profiles of primary initiation for people born in five consecutive decades.** | 8 years | No intervention Observational (Longitudinal)  Snus | N | n/a | Use behavior QA:  Smoking and snus prevalence (current and past); Initiation by Smoking or snus?; time to first CC/snus after waking; CPD, Snus per day; age of initiation of daily smoking / snus; desire to quit; quit attempts; cessation aid used to quit CC (can be snus as well)  Correlation with demographics and the 5 categories of use; profile of initiation  Quitting rates related to initiation profile, age and use behavior | none |
| Rensch 2021 ^98^ | 42 S | USA | RCT 7-way crossover study Nic PK under confinement  Six different flavor variants of nic pouch product  7 day in-clinic with 4h ad lib use session each afternoon and controlled use session each morning (30 min pouch use or CC 10 puffs every 30 sec)  Inclusion: ≥10 CPD ≥1 year with no intention to quit  Control: CC | 7 days | Nicotine pouch on! Different flavors and 4mg nic | Y: Cot (urine): 500ng/mL at screening | Y: at home ad lib prior to study and at site for 30min on day-1 | Subjective effects (QAs):  QSU (Questionnaire on Smoking Urges)  TNW (Tobacco Product/Nicotine Withdrawal VAS)  mCEQ (Modifed Cigarette Evaluation Questionnaire)  DEP (Direct Effects of Product VAS)  Use the Product Again VAS  Use behavior during ad lib sessions | Nic PK |
| Round 2019 ^99^ | 153 S | USA | RCT switching study o EC or nic gum under confinement  2day BL period of ad lib smoking prior to switching to randomized product  5 days ad lib test product use + Nic PK on Day 6  Inclusion: ≥10 CPD having their 1^st^ CC within 30 min of waking  Control: Nic gum as comparator and CC as BL and Nic PK | 7 days | EC Vuse solo 1^st^ gen EC (cigalike)  Tobacco and menthol flavor 48mg/mL Nic  EC vs NRT | N | N | Use behavior:  CPD; gum pieces/d; liquid/day | BoE (urine)  TNE  SPMA, 3-HPMA, MHBMA, HMPMA, CEMA, HEMA, AAMA, GAMA, NNAL, NNN, NAB, NAT, 1-AN, 2-AN, 3-ABP, 4-ABP, o-tol, 1-Nap, 2-Nap, 1-OH-Pyr, 2-OH-Flu, 3-OH-BaP, SCN  COHb  Nic PK of test product and UB CC (control)  BoPH: Urine mutagenicity |
| Ruther 2021 ^100^ | 80 S  Only 49 at follow-up 3months | Germany | Interventional switching study  60 subjects switch to EC and 20 to complete cessation  Subjects shall be willing/motivated to switch but are allowed to continue CC  **Recruitment in vape shop of customers buying an EC for 1^st^ time**  2 Visits: BL and 3 months  Inclusion: ≥10 CPD ≥5 years  Control: Cessation/complete abstinence | 3 months | EC | Y: eCO and Cot (urine) | N | Smoking history  Use behavior (CC + EC); CPD (interview at BL and after 3m)  FTND | FeNO  Spirometry: FEV1, FVC, PEF, FEF25, FEF50, FEF75, FEF25-75  Mannitol provocation test for bronchial activity  Quality of life – short version (WHOQOL-BREF) |
| Sakaguchi 2014 ^101^ | 70 S | Japan | RCT Switching study under confinement  2:1 randomization into HTP and CC (reference cig) group (continue S) in investigational period, diet-controlled **under confinement for 28d**  **Product use controlled for product/d and eight puffs per CC / stick**  4 time points for biospecimen collection: BL, Week 1, 2, 4  Inclusion: ≥20 CPD ≥1 years  Control: Continue CC a reference cigarette | 1 month | HTP Prototype product | Y: Cot (serum) 29ng/mL at screening |  | CPD; Sticks per day | BoE: Blood COHb, plasma SCN, TNE, NNAL, 4-ABP, SPMA, MHBMA, t,t-MA, HMPMA, 3-HPMA, 1-OH-Pyr  Urine mutagenicity |
| Sakaguchi 2021 ^102^ | 459 subjects | Japan | Observational, cross-sectional post-market surveillance study  259 EC users, 100 CC, 100 NS  2 visits: Screening + survey day  Inclusion: Self-identified exclusive EC or CC user or NS  Exclusive: daily on 5+ days a week  Control: CC and NS | 1 day | No intervention observational post-market (Cross-sectional)  EC | Y: eCO (10ppm) and Cot (urine) 200ng/mL at screening | n/a | Use behavior: CPD, capsules/day; Period of use, frequency of use  Use history before EC product use: CPD | BoE: NNAL  Cot (plasma)  BoPH:  Total cholesterol, LDL, HDL, triglyceride, sICAM, WBC, 11-dh-TXB2, 2,3-d-TXB2, 8-i-PGF2a  Respiratory endpoints:  FEV1, FVC, FEF25-75, PEF  Leicester cough QA (LCQ) Quality of Life (SF-36 QA)  composite three component summary score [Physical component summary (PCS),  Mental-component summary (MCS), Role-social-component  summary (RCS)] |
| Shahab 2017 ^103^ | 181 participants | UK | Cross-sectional study with 5 groups  Exclusive CC (≥5CPD ≥6months), Former S with long-term EC or NRT-only use (at least weekly for ≥6months), DU of CC with EC or NRT (at least weekly for ≥6months)  Control: CC | 1 day (single session) | No intervention observational (Cross-sectional)  EC / NRT | Y: eCO (10ppm) | n/a | Use behavior: current and past CPD  Age of smoking initiation, | BoE:  TNE (urine), Nic/Cot (saliva)  NNAL, AAMA, CEMA, MHBMA, HEMA  BoPH:  C-reactive protein (CRP) |
| Sharan 2020 ^104^ | 3000 Vapers | India | Cross-sectional study to assess patterns of tobacco and EC use in India  Interview based survey in current EC users  Inclusion: Current EC users  No controls | 1 assessment | No intervention observational (Cross-sectional)  EC | N | n/a | Interview-based QA:  Use behavior: Past and current smoking and smokeless tobacco use pattern (past: before EC initiation; current: after EC initiation)  FTND  EC use behavior: EC/d, frequency of use, type of products used  Quit by EC initiation: “Did you manage to reduce or quit smoking tobacco with the help of e-cigarettes?” and “Did you manage to reduce or quit chewing (oral) tobacco with the help of e-cigarette?” with responses yes, or yes, I reduced, relapse, no, no+increase in CC  **Risk perception question** | Self-reported changes in health and side effects of EC initiation |
| Shiffman 2021 ^105^ | 55414 subjects | USA | Naturalistic, interventional study  Subjects included irrespective of baseline smoking status  Subjects who purchased JUUL starter kit on own will were asked to participate in study | 1 year | EC JUUL | See Goldenson 2021 | See Goldenson 2021 | See Goldenson 2021 | See Goldenson 2021 |
| Sibul 2021 ^106^ | 60 subjects | Germany | Cross-sectional study in 5 different product use groups of exclusive, experienced users (≥6months exclusive use) of: CC (≥10CPD), EC (≥100puffs/d), HTP (≥10 sticks/d for ≥3months), OT (≥1.5g pouch or 4g loose OT for ≥3months), NRT (≥1month)  76h confinement, diet-controlled  Blood, saliva, urine, EB, EBC collection each day (3 days in-clinic)  Controls: CC and NS | 3 days | No intervention Observational (Cross-sectional)  EC / HTP / Smokeless and pouch / NRT UB for each product type | Y: eCO (7ppm) and Cotinine (urine) 200ng/mL | n/a | Use behavior | BoE  BoPH  Non-targeted screening |
| Song 2020 ^107^ | 73 subjects | USA | Cross-sectional study in 3 groups: CC, EC, NS  **Inclusion: Criteria not reported for EC and CC**  Controls: CC, NS | n/a | No intervention cross-sectional  EC | Y: Cot (saliva) | n/a | Use behavior: Years of use, puffs/d, liquid/d, nic content in e-liquid | Transcriptomics, DNA methylation  Inflammatory cell counts  Cytokines |
| Sreeramareddy 2022 ^108^ | ? | China, Costa Rica, Ethiopia, India, Kaz, Mexico, Philippines, Romania, Russia, Senegal, Ukraine, Turkey, Uruguay, Vietnam | Global Adult Tobacco Survey data in LMICs on awareness about ECs use among adults | n/a | No intervention Observational (Cross-sectional)  EC | N | n/a | Prevalence of awareness  Association of use with CC and sociodemographics | none |
| St Helen 2020 ^109^ | 36 DU | USA | 2-arm crossover RCT in DU asked to use CC or EC exclusively for 7 days each  Visit at BL followed by 4 day ad lib at home use (uncontrolled) and 3 days of confinement (Day5-7). Nic PK on D5 and ad lib use of 1 exclusive product in-clinic for 2days  Inclusion: ≥5 CPD ≥1month and same EC ≥1use/d on 15 of past 30d with nic ≥6mg/mL and no intention to quit CC or EC  Control: CC, abstinence for 2d in-clinic | 7days | EC (UB) | Y: eCO (5ppm) Cot (saliva) 50ng/mL | N: use of UB | Use behavior and subjective measures | BoE:  2-HPMA, 3-HPMA, AAMA, CEMA, HEMA, HMPMA, MHBMA, MMA, NNAL, NDMA, SPMA  Nic PK |
| Strasser 2016 ^110^ | 28 S | USA | RCT in 3 sessions  Day1: UB CC  Day5: EC use  Day 10: EC use  3 Visits in-clinic: Day1, 5, 10  Randomization to 1 of 5 EC brands after Day1 UB CC session. Provision of test EC for Days2-10  Inclusion: ≥10 CPD with no intention to quit | 10 days | EC NJOY 18mgnic; V2 18mg Nic; Green Smoke 20mg nic; blu 20-24mg nic; white cloud 23-24mg nic | Y: eCO (10ppm) and Cot (Saliva) | N | Smoking history  Smoking behavior: CPD; FTND  Withdrawal symptoms and cravings: Withdrawal symptoms checklist; Likert-style scale  Craving: 10-item QA of Smoking urges (QSU-B)  Satisfaction and liking VAS  Use topography (daily diary from D2 – 10):  total no of puffs; puff duration; interpuff interval; total time of use in 10min ad lib session; no of vaping episodes per day | BoE:  Cot (saliva)  eCO |
| Tattan-Birch 2023 ^111^ | 92 S | England, UK | RCT 2-group pragmatic randomized controlled trial  Conducted in stop smoking services in England  Recruitment of S who are willing and trying to quit  Randomized into EC+varenicline and varenicline alone for cessation  S were asked to set target quit date at BL within 1-4 weeks  Weekly support until 12 weeks after quitting  Inclusion: S of CC with the intention to quit | 3 months | EC Aspire EC selected by parrticipants in 3 flavours and 3 nic strengths (6, 12, 18mg/mL)  Participants encouraged to buy further e-liquid  EC vs EC+varenicline | Y: eCO (10ppm) at week 12 (end of study) |  | Smoking abstinence at weeks 9-12 from target quit date  Smoking abstinence at weeks 2-4 from target quit date  Length of abstinence before relapse  Smoking status (CPD) | Respiratory symptoms, i.a. cough |
| Tran 2020 ^112^ | 80 S | Poland | RCT two-arm switching study under confinement  Randomization to HTP test product or continue CC (1:1)  Sample collection at BL and Day5  Admission to clinic 3 days prior to BL assessment  Inclusion: ≥10 CPD ≥3years no intention to quit  Control: Continue CC | 8 days | HTP in 2 nic strengths of 0.5 and 1.3 mg yield (Health Canada Intense regimen) | N | N | FTND  Human Puffing topography by SODIM SPA/M device and Sticks/d; CPD: puff volume, puff duration, no of puffs, puff frequency | BoE:  COHb, eCO  MHBMA, 3-HPMA, SPMA, CEMA, HEMA, HMPMA, NNAL, NNN, 3-OH-BaP, 1-OH-Pyr, o-tol, 1-AN, 2-AN, 4-ABP  TNE, Nic, Cot (plasma)  Urine mutagenicity (Ames)  CYP1A2 activity |
| Tseng 2016 ^113^ | 99 S | USA | RCT two-arm switching study  1:1 randomization to EC or placebo EC (no nic)  Participants encouraged to replace CC with as much or as little EC as needed to reduce nicotine withdrawal symptoms  3 visits: BL, weeks 1, 3  Inclusion: ≥10 CPD willing to reduce CPD  Control: None; only comparator placebo EC but no CC or NS group | 3 weeks | EC: NJOY tobacco flavour 45mg/mL Nic  Placebo EC with same flavour but no Nic | Y: eCO (8ppm) | N | Self-reported reduction of at least 50% in CPD  % reduction in CPD  7-day point prevalence abstinence (verified by eCO)  EC use  Satisfaction with EC: single question on 5-pt Likert scale  Smoking cessation self-efficacy was assessed by a single question  Readiness to quit :Readiness to Quit Ladder. | none |
| Veldheer 2019 ^114^ | 263 S | USA | RCT 4-arm to encourage participants to reduce CPD by EC or non-electronic cig substitute  1-arm was cig-sub; arm 2-4: EC in the different nic strengths  6 visits: BL, weeks 1, 2, 4, 8, 12  Assessments only at BL and weeks 4, 12  Inclusion: ≥10 CPD willing to reduce their CPD  Control: None; only placebo cig-substitute in one arm | 3 months | EC SmockTech in 3 nic strengths (0, 8, 36mg/mL)  Cig-substitute: QuitSmart Inc: does not contain nic or emit aerosol | N | N | Penn State Cigarette Dependence Index (PSCDI)  Product use  Reduction in CPD in past 7 days at 1m, 3m | eCO  Spirometry (FEV1, FVC, FEF25-75, FET)  Blood pressure, pulse |
| Voos 2019 ^115^ | 18 S | USA | RCT within-subject crossover design  8 visits/sessions: at visit 1 screening, v2: UB CC ad lib Nic PK; visits 3-7 controlled puffing sessions with test products and Nic PKs  Inclusion: ≥10 CPD  Control: CC session | 8 weeks | EC 6 different products disposable v2, rechargeable Green Smoke, eGO v2, mod iTazte, e-cigar Cuvana, e-pipe Smoktech  Nic strength 18 and 24mg/mL  Product 1 and 2 early gen cigalike  3 and 4 new gen EC | Y: eCO (8ppm) at each visit | Y: practice with test devices at home for 1week prior to use session | Product ranking QA  Subjective effects QA  Device satisfaction QA  At end (visit 8): Product ranking QA  Nicotine withdrawal symptoms: Minnesota Nicotine Withdrawal Scale (MNWS)  Craving to Smoke: QA of Smoking Urges-Brief (QSU-B)  Subjective effects of nicotine: Drug Effect QA (DEQ-5)  Satisfaction (Liking): 12-item adapted evaluation scale | Nic PK |
| Walele 2018 ^116^ | 206 S  102 completers at 21m | UK | Ambulatory, interventional clinical trial with same subjects as Cravo 2016 (Cravo was RCT)  All subjects switch to using study EC  11 Visits: BL, months 1, 2, 3, 6, 9, 12, 15, 18, 21  Inclusion: 5-30 CPD for ≥1year with no intention to quit S  Control: None | 21 months | EC Puritane closed system EC (cigalike 1^st^ gen)  16mg/mL Nic  Tobacco flavored used first 3m  From m3 chose between tobacco and menthol | Y: eCO (6ppm) | Y: session 2weeks before BL and training at BL | Nicotine withdrawal symptoms MNWS  Urge to smoke QSU-B  Use behavior  See Cravo 2016 | AE  12-lead ECG  Lung function tests  BoE  See Cravo 2016 |
| Walker 2020 ^117^ | 1125 S | New Zealand | RCT subjects randomized to nic patch, patch+EC 18mgNic, patch+EC 0mg Nic  Ad lib use of EC. Subjects shall quit at their quit date and use EC 2w prior to quit date. Use of EC at least for 12weeks after quitting  Visits/Outcomes: Quit date, months 1, 3, 6, 12  Inclusion: S with the intention to quit in next 2weeks  Control: nic patch as comparator | 12 months | EC Refillable 2^nd^ gen Kangertech 18mg/mL Nic or 0mg/mL  Tobacco flavor | Y: eCO | N | Continuous abstinence at 6m  Relapse  Tobacco withdrawal symptoms  urge to smoke  Urge to vape  Self-reported weight  Treatment cross-over  Continued use of allocated treatment past 14 weeks  Belief in ability to quit and remain tobacco-free  Smoking identity and views on their allocated treatment for smoking cessation and whether they would recommend it to other people who smoke who want to quit  In people still smoking at each follow-up call, CPD and reduction in smoking  Participants allocated e-cigarettes were asked about their urge to vape; whether they changed devices or e-liquid, or both; whether they accessed any e-cigarette support | Changes in shortness of breath, cough, asthma, COPD, and mental health problems |
| Yoon 2022 ^118^ | 19389 adults  3929 recent S | South Korea | Longitudinal study 7^th^ Korea National Health and Nutrition Examination (KNHANES) 2016 to 2018  Subcohort of 3929 S was used to examine relationship between EC use and smoking cessation  Current S: more than 100 CC lifetime and currently S every day or some days  Former S: more than 100 CC lifetime and currently S not at all and quit in past 2years | n/a | No intervention observational (Longitudinal)  EC | N | n/a | CPD  Use behavior: EC use: never, current, former  CC quitting: past-year quit attempts, intention to quit, duration of quitting  **Stages of change in smoking cessation:**  1) Precontemplation (PC): current S who are not planning on quitting within the next 6 months; 2) Contemplation (C): current S who are planning on quitting within the next 6 months; 3) Preparation (P): current S who are both planning on quitting within the next month and have quit attempts in the past year; 4) Action (A): recent quitters who quit within the last 6 months; 5) Maintenance (M): recent quitters who quit more than 6 months ago | none |
| Yuki 2018 ^119^ | 60 S | Japan | RCT 3-arm single center switching study under confinement  Randomization to EC, continue CC or cessation (abstinence) 1:1:1  Check-in evening of Day-2. Day-1 CC according to self-reported CPD. Day 1-5 switch to EC or CC or cessation. Ad lib use of EC but limited to 10 capsules/day to reflect usual CC. CC group ad lib within +-10% of their usual CPD  BoE Assessments BL, D3, D5  Inclusion: ≥11 CPD for ≥1year  Control: Continue CC and cessation | 5 days | EC tobacco flavored | Y: Cot (urine) | N | Product use: CPD, Capsules/d  Puffing topography by CReSSmicro (Borgwaldt):  No. of puffs, puff volume, puff duration | BoE:  3-HPMA, CEMA, SPMA, MHBMA, HMPMA, HEMA, 4-ABP, 1-AN, 2-AN, o-tol, 3-OH-BaP, 1-OH-Pyr, NNAL, NNN, eCO |
| Zhao ^120^ | 189306 adults from 2015 and 184475 adults from 2018 | China | Assessment based on 2 nationally representative cross-sectional surveys from the China Chronic Disease and Nutrition Surveillance (CCDNS) in 2015 and 2018  Past 30d EC users:1+d last month  Frequent EC users: 20+d last month  Current S classified into daily and occasional | 3 years | none observational (Longitudinal)  EC | N | n/a | CPD  Risk perception  EC use prevalence | none |

**References**

1. Adriaens K, Van Gucht D, Declerck P and Baeyens F. Effectiveness of the Electronic Cigarette: An Eight-Week Flemish Study with Six-Month Follow-up on Smoking Reduction, Craving and Experienced Benefits and Complaints. *Int J Environ Res Public Health*. 2014; 11: 11220-48. doi: 10.3390/ijerph111111220.

2. Azagba S, Qeadan F, Shan L, Latham K and Wolfson M. E-Cigarette Use and Transition in Adult Smoking Frequency: A Longitudinal Study. *Am J Prev Med*. 2020; 59: 367-76.

3. Azzopardi D, Ebajemito J, McEwan M, et al. A randomised study to assess the nicotine pharmacokinetics of an oral nicotine pouch and two nicotine replacement therapy products. *Sci Rep*. 2022; 12: 6949.

4. Azzopardi D, Haswell LE, Frosina J, et al. Biomarkers of Exposure and Potential Harm in Exclusive Users of Nicotine Pouches and Current, Former, and Never Smokers: Protocol for a Cross-sectional Clinical Study. *JMIR research protocols*. 2022; 11: e39785.

5. Baldassarri SR, Bernstein SL, Chupp GL, Slade MD, Fucito LM and Toll BA. Electronic cigarettes for adults with tobacco dependence enrolled in a tobacco treatment program: A pilot study. *Addict Behav*. 2018; 80: 1-5.

6. Bell S, Dean J, Gilks C, et al. Tobacco Harm Reduction with Vaporised Nicotine (THRiVe): The Study Protocol of an Uncontrolled Feasibility Study of Novel Nicotine Replacement Products among People Living with HIV Who Smoke. *Int J Environ Res Public Health*. 2017; 14.

7. Berenguer C, Pereira JAM and Câmara JS. Urinary volatomic profile of traditional tobacco smokers and electronic cigarettes users as a strategy to unveil potential health issues. *J Sep Sci*. 2022; 45: 582-93.

8. Blank ML, Hoek J, George M, et al. An Exploration of Smoking-to-Vaping Transition Attempts Using a "Smart" Electronic Nicotine Delivery System. *Nicotine Tob Res*. 2019; 21: 1339-46.

9. Burris JL, Carpenter MJ, Wahlquist AE, Cummings KM and Gray KM. Brief, instructional smokeless tobacco use among cigarette smokers who do not intend to quit: a pilot randomized clinical trial. *Nicotine Tob Res*. 2014; 16: 397-405.

10. Camacho OM, Shepperd CJ, Eldridge A, Meyer I and Proctor CJ. Reference change values to assess changes in concentrations of biomarkers of exposure in individuals participating in a cigarette-switching study. *Clin Chem Lab Med*. 2014; 52: 399-411.

11. Caponnetto P, Auditore R, Russo C, Cappello GC and Polosa R. Impact of an electronic cigarette on smoking reduction and cessation in schizophrenic smokers: a prospective 12-month pilot study. *Int J Environ Res Public Health*. 2013; 10: 446-61.

12. Caponnetto P, Campagna D, Cibella F, et al. EffiCiency and Safety of an eLectronic cigAreTte (ECLAT) as Tobacco Cigarettes Substitute: A Prospective 12-Month Randomized Control Design Study. *PloS one*. 2013; 8: e66317.

13. Caponnetto P, Caruso M, Maglia M, et al. Non-inferiority trial comparing cigarette consumption, adoption rates, acceptability, tolerability, and tobacco harm reduction potential in smokers switching to Heated Tobacco Products or electronic cigarettes: Study protocol for a randomized controlled trial. *Contemp Clin Trials Commun*. 2020; 17: 100518.

14. Caponnetto P, Maglia M, Prosperini G, Busà B and Polosa R. Carbon monoxide levels after inhalation from new generation heated tobacco products. *Respir Res*. 2018; 19: 164.

15. Carpenter MJ, Heckman BW, Wahlquist AE, et al. A Naturalistic, Randomized Pilot Trial of E-Cigarettes: Uptake, Exposure, and Behavioral Effects. *Cancer Epidemiol Biomarkers Prev*. 2017; 26: 1795-803.

16. Chapman F, McDermott S, Rudd K, et al. A randomised, open-label, cross-over clinical study to evaluate the pharmacokinetic, pharmacodynamic and safety and tolerability profiles of tobacco-free oral nicotine pouches relative to cigarettes. *Psychopharmacology (Berl)*. 2022; 239: 2931-43.

17. Choi S, Lee K and Park SM. Combined Associations of Changes in Noncombustible Nicotine or Tobacco Product and Combustible Cigarette Use Habits With Subsequent Short-Term Cardiovascular Disease Risk Among South Korean Men: A Nationwide Cohort Study. *Circulation*. 2021; 144: 1528-38.

18. Cobb CO, Foulds J, Yen MS, et al. Effect of an electronic nicotine delivery system with 0, 8, or 36 mg/mL liquid nicotine versus a cigarette substitute on tobacco-related toxicant exposure: a four-arm, parallel-group, randomised, controlled trial. *Lancet Respir Med*. 2021; 9: 840-50.

19. Cohen G, Goldenson NI, Bailey PC, Chan S and Shiffman S. Changes in Biomarkers of Cigarette Smoke Exposure After 6 Days of Switching Exclusively or Partially to Use of the JUUL System with Two Nicotine Concentrations: A Randomized Controlled Confinement Study in Adult Smokers. *Nicotine Tob Res*. 2021; 23: 2153-61.

20. Cravo AS, Bush J, Sharma G, et al. A randomised, parallel group study to evaluate the safety profile of an electronic vapour product over 12 weeks. *Regul Toxicol Pharmacol*. 2016; 81 Suppl 1: S1-S14.

21. Czoli CD, Fong GT, Goniewicz ML and Hammond D. Biomarkers of exposure among "dual users" of tobacco cigarettes and electronic cigarettes in Canada. *Nicotine & tobacco research : official journal of the Society for Research on Nicotine and Tobacco*. 2019; 21: 1259-66.

22. D'Ruiz CD, Graff DW and Yan XS. Nicotine delivery, tolerability and reduction of smoking urge in smokers following short-term use of one brand of electronic cigarettes. *BMC Public Health*. 2015; 15: 991.

23. D'Ruiz CD, O'Connell G, Graff DW and Yan XS. Measurement of cardiovascular and pulmonary function endpoints and other physiological effects following partial or complete substitution of cigarettes with electronic cigarettes in adult smokers. *Regul Toxicol Pharmacol*. 2017; 87: 36-53.

24. Ebajemito JK, McEwan M, Gale N, Camacho OM, Hardie G and Proctor CJ. A randomised controlled single-centre open-label pharmacokinetic study to examine various approaches of nicotine delivery using electronic cigarettes. *Sci Rep*. 2020; 10: 19980.

25. Edmiston JS, Webb KM, Wang J, Oliveri D, Liang Q and Sarkar M. Biomarkers of Exposure and Biomarkers of Potential Harm in Adult Smokers Who Switch to e-Vapor Products Relative to Cigarette Smoking in a 24-week, Randomized, Clinical Trial. *Nicotine Tob Res*. 2022; 24: 1047-54.

26. Eisenberg MJ, Hebert-Losier A, Windle SB, et al. Effect of e-Cigarettes Plus Counseling vs Counseling Alone on Smoking Cessation: A Randomized Clinical Trial. *JAMA*. 2020; 324: 1844-54.

27. Fearon IM, Eldridge A, Gale N, et al. E-cigarette Nicotine Delivery: Data and Learnings from Pharmacokinetic Studies. *Am J Health Behav*. 2017; 41: 16-32.

28. Feng J, Sosnoff CS, Bernert JT, et al. Urinary Nicotine Metabolites and Self-Reported Tobacco Use Among Adults in the Population Assessment of Tobacco and Health (PATH) Study, 2013-2014. *Nicotine Tob Res*. 2022; 24: 768-77.

29. Ferrari M, Zanasi A, Nardi E, et al. Short-term effects of a nicotine-free e-cigarette compared to a traditional cigarette in smokers and non-smokers. *BMC Pulm Med*. 2015; 15: 120.

30. Flacco ME, Fiore M, Acuti Martellucci C, et al. Tobacco vs. electronic cigarettes: absence of harm reduction after six years of follow-up. *Eur Rev Med Pharmacol Sci*. 2020; 24: 3923-34.

31. Fraser D, Borland R and Gartner C. Protocol for a randomised pragmatic policy trial of nicotine products for quitting or long-term substitution in smokers. *BMC Public Health*. 2015; 15: 1026.

32. Fu R, O'Connor S, Diemert L, et al. Real-world vaping experiences and smoking cessation among cigarette smoking adults. *Addict Behav*. 2021; 116: 106814.

33. Gale N, McEwan M, Eldridge AC, et al. Changes in Biomarkers of Exposure on Switching From a Conventional Cigarette to Tobacco Heating Products: A Randomized, Controlled Study in Healthy Japanese Subjects. *Nicotine Tob Res*. 2019; 21: 1220-7.

34. Gale N, McEwan M, Hardie G, Proctor CJ and Murphy J. Changes in biomarkers of exposure and biomarkers of potential harm after 360 days in smokers who either continue to smoke, switch to a tobacco heating product or quit smoking. *Intern Emerg Med*. 2022; 17: 2017-30.

35. George J, Hussain M, Vadiveloo T, et al. Cardiovascular Effects of Switching From Tobacco Cigarettes to Electronic Cigarettes. *J Am Coll Cardiol*. 2019; 74: 3112-20.

36. Gmel G, Baggio S, Mohler-Kuo M, Daeppen JB and Studer J. E-cigarette use in young Swiss men: is vaping an effective way of reducing or quitting smoking? *Swiss Med Wkly*. 2016; 146: w14271.

37. Goldenson NI, Shiffman S, Hatcher C, et al. Switching away from Cigarettes across 12 Months among Adult Smokers Purchasing the JUUL System. *Am J Health Behav*. 2021; 45: 443-63.

38. Goniewicz ML, Gawron M, Smith DM, Peng M, Jacob P, 3rd and Benowitz NL. Exposure to Nicotine and Selected Toxicants in Cigarette Smokers Who Switched to Electronic Cigarettes: A Longitudinal Within-Subjects Observational Study. *Nicotine Tob Res*. 2017; 19: 160-7.

39. Gorini G, Ferrante G, Quarchioni E, et al. Electronic cigarette use as an aid to quit smoking in the representative Italian population PASSI survey. *Prev Med*. 2017; 102: 1-5.

40. Guttentag A, Tseng TY, Shelley D and Kirchner T. Analyzing Trajectories of Acute Cigarette Reduction Post-Introduction of an E-Cigarette Using Ecological Momentary Assessment Data. *Int J Environ Res Public Health*. 2022; 19.

41. Hajek P, Phillips-Waller A, Przulj D, et al. A Randomized Trial of E-Cigarettes versus Nicotine-Replacement Therapy. *N Engl J Med*. 2019; 380: 629-37.

42. Hajek P, Przulj D, Pesola F, et al. Electronic cigarettes versus nicotine patches for smoking cessation in pregnancy: a randomized controlled trial. *Nat Med*. 2022; 28: 958-64.

43. Han DH, Lee SH and Seo DC. Within-Person Longitudinal Associations Between Electronic Nicotine Delivery Systems Use and Smoking Cessation Efforts Among US Continuing Adult Cigarette Smokers. *Nicotine Tob Res*. 2022; 24: 590-7.

44. Harada S, Sata M, Matsumoto M, et al. Changes in Smoking Habits and Behaviors Following the Introduction and Spread of Heated Tobacco Products in Japan and Its Effect on FEV(1) Decline: A Longitudinal Cohort Study. *J Epidemiol*. 2022; 32: 180-7.

45. Hardie G, Gale N, McEwan M, et al. An abuse liability assessment of the glo tobacco heating product in comparison to combustible cigarettes and nicotine replacement therapy. *Sci Rep*. 2022; 12: 14701.

46. Harlow AF, Fetterman JL, Ross CS, et al. Association of device type, flavours and vaping behaviour with tobacco product transitions among adult electronic cigarette users in the USA. *Tob Control*. 2022; 31: e10-e7.

47. Hatsukami DK, Meier E, Lindgren BR, et al. A Randomized Clinical Trial Examining the Effects of Instructions for Electronic Cigarette Use on Smoking-Related Behaviors and Biomarkers of Exposure. *Nicotine Tob Res*. 2020; 22: 1524-32.

48. Hatsukami DK, Severson H, Anderson A, et al. Randomised clinical trial of snus versus medicinal nicotine among smokers interested in product switching. *Tob Control*. 2016; 25: 267-74.

49. Haziza C, de La Bourdonnaye G, Donelli A, et al. Favorable Changes in Biomarkers of Potential Harm to Reduce the Adverse Health Effects of Smoking in Smokers Switching to the Menthol Tobacco Heating System 2.2 for 3 Months (Part 2). *Nicotine Tob Res*. 2020; 22: 549-59.

50. Haziza C, de La Bourdonnaye G, Merlet S, et al. Assessment of the reduction in levels of exposure to harmful and potentially harmful constituents in Japanese subjects using a novel tobacco heating system compared with conventional cigarettes and smoking abstinence: A randomized controlled study in confinement. *Regul Toxicol Pharmacol*. 2016; 81: 489-99.

51. Haziza C, de La Bourdonnaye G, Skiada D, et al. Evaluation of the Tobacco Heating System 2.2. Part 8: 5-Day randomized reduced exposure clinical study in Poland. *Regul Toxicol Pharmacol*. 2016; 81 Suppl 2: S139-S50.

52. Ikonomidis I, Katogiannis K, Kostelli G, et al. Effects of electronic cigarette on platelet and vascular function after four months of use. *Food Chem Toxicol*. 2020; 141: 111389.

53. Ikonomidis I, Vlastos D, Kourea K, et al. Electronic Cigarette Smoking Increases Arterial Stiffness and Oxidative Stress to a Lesser Extent Than a Single Conventional Cigarette: An Acute and Chronic Study. *Circulation*. 2018; 137: 303-6.

54. Jankowski M, Ostrowska A, Sierpinski R, et al. The Prevalence of Tobacco, Heated Tobacco, and E-Cigarette Use in Poland: A 2022 Web-Based Cross-Sectional Survey. *Int J Environ Res Public Health*. 2022; 19.

55. Jay J, Pfaunmiller EL, Huang NJ, Cohen G and Graff DW. Five-Day Changes in Biomarkers of Exposure Among Adult Smokers After Completely Switching From Combustible Cigarettes to a Nicotine-Salt Pod System. *Nicotine & Tobacco Research*. 2019; 22: 1285-93.

56. Kaplan B, Galiatsatos P, Breland A, Eissenberg T and Cohen JE. Effectiveness of ENDS, NRT and medication for smoking cessation among cigarette-only users: a longitudinal analysis of PATH Study wave 3 (2015-2016) and 4 (2016-2017), adult data. *Tob Control*. 2021.

57. Kimber CF, Soar K and Dawkins LE. Changes in puffing topography and subjective effects over a 2-week period in e-cigarette naive smokers: Effects of device type and nicotine concentrations. *Addict Behav*. 2021; 118: 106909.

58. Kotz D, Jackson S, Brown J and Kastaun S. The Effectiveness of E-Cigarettes for Smoking Cessation. *Dtsch Arztebl Int*. 2022; 119: 297-301.

59. Krautter GR, Chen PX and Borgerding MF. Consumption patterns and biomarkers of exposure in cigarette smokers switched to Snus, various dissolvable tobacco products, Dual use, or tobacco abstinence. *Regul Toxicol Pharmacol*. 2015; 71: 186-97.

60. Krishnan N, Abroms LC and Berg CJ. Electronic Nicotine Product Cessation and Cigarette Smoking: Analysis of Waves 3 and 4 From the PATH Study. *Nicotine Tob Res*. 2022; 24: 324-32.

61. Kumral TL, Salturk Z, Yildirim G, et al. How does electronic cigarette smoking affect sinonasal symptoms and nasal mucociliary clearance? *B-ent*. 2016; 12: 17-21.

62. Lechner WV, Meier E, Wiener JL, et al. The comparative efficacy of first- versus second-generation electronic cigarettes in reducing symptoms of nicotine withdrawal. *Addiction*. 2015; 110: 862-7.

63. Lee SH, Ahn SH and Cheong YS. Effect of Electronic Cigarettes on Smoking Reduction and Cessation in Korean Male Smokers: A Randomized Controlled Study. *J Am Board Fam Med*. 2019; 32: 567-74.

64. Li J, Hajek P, Pesola F, et al. Cost-effectiveness of e-cigarettes compared with nicotine replacement therapy in stop smoking services in England (TEC study): a randomized controlled trial. *Addiction*. 2020; 115: 507-17.

65. Liu G, Lin CJ, Yates CR and Prasad GL. Metabolomic Analysis Identified Reduced Levels of Xenobiotics, Oxidative Stress, and Improved Vitamin Metabolism in Smokers Switched to Vuse Electronic Nicotine Delivery System. *Nicotine Tob Res*. 2021; 23: 1133-42.

66. Lucchiari C, Masiero M, Mazzocco K, et al. Benefits of e-cigarettes in smoking reduction and in pulmonary health among chronic smokers undergoing a lung cancer screening program at 6 months. *Addict Behav*. 2020; 103: 106222.

67. Ludicke F, Ansari SM, Lama N, et al. Effects of Switching to a Heat-Not-Burn Tobacco Product on Biologically Relevant Biomarkers to Assess a Candidate Modified Risk Tobacco Product: A Randomized Trial. *Cancer Epidemiol Biomarkers Prev*. 2019; 28: 1934-43.

68. Ludicke F, Baker G, Magnette J, Picavet P and Weitkunat R. Reduced Exposure to Harmful and Potentially Harmful Smoke Constituents With the Tobacco Heating System 2.1. *Nicotine Tob Res*. 2017; 19: 168-75.

69. Luk TT, Weng X, Wu YS, et al. Association of heated tobacco product use with smoking cessation in Chinese cigarette smokers in Hong Kong: a prospective study. *Tob Control*. 2021; 30: 653-9.

70. Manzoli L, Flacco ME, Ferrante M, et al. Cohort study of electronic cigarette use: effectiveness and safety at 24 months. *Tob Control*. 2017; 26: 284-92.

71. Martner SG and Dallery J. Technology-based contingency management and e-cigarettes during the initial weeks of a smoking quit attempt. *J Appl Behav Anal*. 2019; 52: 928-43.

72. McEwan M, Azzopardi D, Gale N, et al. A Randomised Study to Investigate the Nicotine Pharmacokinetics of Oral Nicotine Pouches and a Combustible Cigarette. *Eur J Drug Metab Pharmacokinet*. 2022; 47: 211-21.

73. McRobbie H, Phillips A, Goniewicz ML, et al. Effects of Switching to Electronic Cigarettes with and without Concurrent Smoking on Exposure to Nicotine, Carbon Monoxide, and Acrolein. *Cancer Prev Res (Phila)*. 2015; 8: 873-8.

74. Meier E, Wahlquist AE, Heckman BW, Cummings KM, Froeliger B and Carpenter MJ. A Pilot Randomized Crossover Trial of Electronic Cigarette Sampling Among Smokers. *Nicotine Tob Res*. 2017; 19: 176-82.

75. Meltzer LR, Simmons VN, Sutton SK, et al. A randomized controlled trial of a smoking cessation self-help intervention for dual users of tobacco cigarettes and E-cigarettes: Intervention development and research design. *Contemp Clin Trials*. 2017; 60: 56-62.

76. Miura N, Yuki D, Minami N, Kakehi A and Futamura Y. A study to investigate changes in the levels of biomarkers of exposure to selected cigarette smoke constituents in Japanese adult male smokers who switched to a non-combustion inhaler type of tobacco product. *Regul Toxicol Pharmacol*. 2015; 71: 498-506.

77. Morphett K, Fraser D, Borland R, et al. A Pragmatic Randomized Comparative Trial of e-Cigarettes and Other Nicotine Products for Quitting or Long-Term Substitution in Smokers. *Nicotine Tob Res*. 2022; 24: 1079-88.

78. Morris P, McDermott S, Chapman F, et al. Reductions in biomarkers of exposure to selected harmful and potentially harmful constituents following exclusive and partial switching from combustible cigarettes to myblu() electronic nicotine delivery systems (ENDS). *Intern Emerg Med*. 2022; 17: 397-410.

79. Myers Smith K, Phillips-Waller A, Pesola F, et al. E-cigarettes versus nicotine replacement treatment as harm reduction interventions for smokers who find quitting difficult: randomized controlled trial. *Addiction*. 2022; 117: 224-33.

80. Nides MA, Leischow SJ, Bhatter M and Simmons M. Nicotine blood levels and short-term smoking reduction with an electronic nicotine delivery system. *Am J Health Behav*. 2014; 38: 265-74.

81. O'Connell G, Graff DW and D'Ruiz CD. Reductions in biomarkers of exposure (BoE) to harmful or potentially harmful constituents (HPHCs) following partial or complete substitution of cigarettes with electronic cigarettes in adult smokers. *Toxicol Mech Methods*. 2016; 26: 443-54.

82. O'Connell G, Pritchard JD, Prue C, et al. A randomised, open-label, cross-over clinical study to evaluate the pharmacokinetic profiles of cigarettes and e-cigarettes with nicotine salt formulations in US adult smokers. *Intern Emerg Med*. 2019; 14: 853-61.

83. O'Connor RJ, Lindgren BR, Schneller LM, Shields PG and Hatsukami DK. Evaluating the utility of subjective effects measures for predicting product sampling, enrollment, and retention in a clinical trial of a smokeless tobacco product. *Addict Behav*. 2018; 76: 95-9.

84. Odani S, Tsuno K, Agaku IT and Tabuchi T. Heated tobacco products do not help smokers quit or prevent relapse: a longitudinal study in Japan. *Tob Control*. 2023.

85. Ogden MW, Marano KM, Jones BA and Stiles MF. Switching from usual brand cigarettes to a tobacco-heating cigarette or snus: Part 1. Study design and methodology. *Biomarkers*. 2015; 20: 382-90.

86. Ohmomo H, Harada S, Komaki S, et al. DNA Methylation Abnormalities and Altered Whole Transcriptome Profiles after Switching from Combustible Tobacco Smoking to Heated Tobacco Products. *Cancer Epidemiol Biomarkers Prev*. 2022; 31: 269-79.

87. Oncken CA, Litt MD, McLaughlin LD and Burki NA. Nicotine concentrations with electronic cigarette use: effects of sex and flavor. *Nicotine Tob Res*. 2015; 17: 473-8.

88. Osibogun O, Bursac Z, McKee M, Li T and Maziak W. Cessation outcomes in adult dual users of e-cigarettes and cigarettes: the Population Assessment of Tobacco and Health cohort study, USA, 2013-2016. *Int J Public Health*. 2020; 65: 923-36.

89. Pacifici R, Pichini S, Graziano S, Pellegrini M, Massaro G and Beatrice F. Successful Nicotine Intake in Medical Assisted Use of E-Cigarettes: A Pilot Study. *Int J Environ Res Public Health*. 2015; 12: 7638-46.

90. Pierce JP, Benmarhnia T, Chen R, et al. Role of e-cigarettes and pharmacotherapy during attempts to quit cigarette smoking: The PATH Study 2013-16. *PloS one*. 2020; 15: e0237938.

91. Polosa R, Caponnetto P, Cibella F and Le-Houezec J. Quit and smoking reduction rates in vape shop consumers: a prospective 12-month survey. *Int J Environ Res Public Health*. 2015; 12: 3428-38.

92. Polosa R, Caponnetto P, Maglia M, Morjaria JB and Russo C. Success rates with nicotine personal vaporizers: a prospective 6-month pilot study of smokers no intention to quit. *BMC Public Health*. 2014; 14: 1159.

93. Polosa R, Morjaria JB, Caponnetto P, et al. Effectiveness and tolerability of electronic cigarette in real-life: a 24-month prospective observational study. *Intern Emerg Med*. 2014; 9: 537-46.

94. Price AD, Coffey M, Houston L and Cook PA. Evaluation of a pharmacy supported e-cigarette smoking cessation intervention in Northwest England. *BMC Public Health*. 2022; 22: 1326.

95. Prokopowicz A, Sobczak A, Szdzuj J, Grygoyc K and Kosmider L. Metal Concentration Assessment in the Urine of Cigarette Smokers Who Switched to Electronic Cigarettes: A Pilot Study. *Int J Environ Res Public Health*. 2020; 17.

96. Pulvers K, Nollen NL, Rice M, et al. Effect of Pod e-Cigarettes vs Cigarettes on Carcinogen Exposure Among African American and Latinx Smokers: A Randomized Clinical Trial. *JAMA Netw Open*. 2020; 3: e2026324.

97. Ramstrom L, Borland R and Wikmans T. Patterns of Smoking and Snus Use in Sweden: Implications for Public Health. *Int J Environ Res Public Health*. 2016; 13.

98. Rensch J, Liu J, Wang J, Vansickel A, Edmiston J and Sarkar M. Nicotine pharmacokinetics and subjective response among adult smokers using different flavors of on!(R) nicotine pouches compared to combustible cigarettes. *Psychopharmacology (Berl)*. 2021; 238: 3325-34.

99. Round EK, Chen P, Taylor AK and Schmidt E. Biomarkers of Tobacco Exposure Decrease After Smokers Switch to an E-Cigarette or Nicotine Gum. *Nicotine Tob Res*. 2019; 21: 1239-47.

100. Ruther T, Kahnert K, Mader M, et al. Reduction of bronchial response to mannitol after partial switch from conventional tobacco to electronic cigarette consumption. *Respir Med*. 2021; 178: 106324.

101. Sakaguchi C, Kakehi A, Minami N, Kikuchi A and Futamura Y. Exposure evaluation of adult male Japanese smokers switched to a heated cigarette in a controlled clinical setting. *Regul Toxicol Pharmacol*. 2014; 69: 338-47.

102. Sakaguchi C, Nagata Y, Kikuchi A, Takeshige Y and Minami N. Differences in Levels of Biomarkers of Potential Harm Among Users of a Heat-Not-Burn Tobacco Product, Cigarette Smokers, and Never-Smokers in Japan: A Post-Marketing Observational Study. *Nicotine Tob Res*. 2021; 23: 1143-52.

103. Shahab L, Goniewicz ML, Blount BC, et al. Nicotine, Carcinogen, and Toxin Exposure in Long-Term E-Cigarette and Nicotine Replacement Therapy Users: A Cross-sectional Study. *Ann Intern Med*. 2017; 166: 390-400.

104. Sharan RN, Chanu TM, Chakrabarty TK and Farsalinos K. Patterns of tobacco and e-cigarette use status in India: a cross-sectional survey of 3000 vapers in eight Indian cities. *Harm Reduct J*. 2020; 17: 21.

105. Shiffman S, Sembower MA, Augustson EM, et al. The Adult JUUL Switching and Smoking Trajectories (ADJUSST) Study: Methods and Analysis of Loss-to-Follow-up. *Am J Health Behav*. 2021; 45: 419-42.

106. Sibul F, Burkhardt T, Kachhadia A, et al. Identification of biomarkers specific to five different nicotine product user groups: Study protocol of a controlled clinical trial. *Contemp Clin Trials Commun*. 2021; 22: 100794.

107. Song MA, Freudenheim JL, Brasky TM, et al. Biomarkers of Exposure and Effect in the Lungs of Smokers, Nonsmokers, and Electronic Cigarette Users. *Cancer Epidemiol Biomarkers Prev*. 2020; 29: 443-51.

108. Sreeramareddy CT and Manoharan A. Awareness About and E-Cigarette Use Among Adults in 15 Low- and Middle-Income Countries, 2014-2018 Estimates From Global Adult Tobacco Surveys. *Nicotine Tob Res*. 2022; 24: 1095-103.

109. St Helen G, Liakoni E, Nardone N, Addo N, Jacob P, 3rd and Benowitz NL. Comparison of Systemic Exposure to Toxic and/or Carcinogenic Volatile Organic Compounds (VOC) during Vaping, Smoking, and Abstention. *Cancer Prev Res (Phila)*. 2020; 13: 153-62.

110. Strasser AA, Souprountchouk V, Kaufmann A, et al. Nicotine Replacement, Topography, and Smoking Phenotypes of E-cigarettes. *Tobacco regulatory science*. 2016; 2: 352-62.

111. Tattan-Birch H, Kock L, Brown J, et al. E-cigarettes to Augment Stop Smoking In-person Support and Treatment With Varenicline (E-ASSIST): A Pragmatic Randomized Controlled Trial. *Nicotine Tob Res*. 2023; 25: 395-403.

112. Tran CT, Bosilkovska M, de La Bourdonnaye G, Blanc N and Haziza C. Reduced levels of biomarkers of exposure in smokers switching to the Carbon-Heated Tobacco Product 1.0: a controlled, randomized, open-label 5-day exposure trial. *Sci Rep*. 2020; 10: 19227.

113. Tseng TY, Ostroff JS, Campo A, et al. A Randomized Trial Comparing the Effect of Nicotine Versus Placebo Electronic Cigarettes on Smoking Reduction Among Young Adult Smokers. *Nicotine Tob Res*. 2016; 18: 1937-43.

114. Veldheer S, Yingst J, Midya V, et al. Pulmonary and other health effects of electronic cigarette use among adult smokers participating in a randomized controlled smoking reduction trial. *Addict Behav*. 2019; 91: 95-101.

115. Voos N, Kaiser L, Mahoney MC, et al. Randomized within-subject trial to evaluate smokers' initial perceptions, subjective effects and nicotine delivery across six vaporized nicotine products. *Addiction*. 2019; 114: 1236-48.

116. Walele T, Bush J, Koch A, Savioz R, Martin C and O'Connell G. Evaluation of the safety profile of an electronic vapour product used for two years by smokers in a real-life setting. *Regul Toxicol Pharmacol*. 2018; 92: 226-38.

117. Walker N, Parag V, Verbiest M, Laking G, Laugesen M and Bullen C. Nicotine patches used in combination with e-cigarettes (with and without nicotine) for smoking cessation: a pragmatic, randomised trial. *Lancet Respir Med*. 2020; 8: 54-64.

118. Yoon W, Cho I and Cho SI. Understanding the role of e-cigarette use in smoking cessation based on the stages of change model. *PloS one*. 2022; 17: e0274311.

119. Yuki D, Takeshige Y, Nakaya K and Futamura Y. Assessment of the exposure to harmful and potentially harmful constituents in healthy Japanese smokers using a novel tobacco vapor product compared with conventional cigarettes and smoking abstinence. *Regul Toxicol Pharmacol*. 2018; 96: 127-34.

120. Zhao Z, Zhang M, Wu J, et al. E-cigarette use among adults in China: findings from repeated cross-sectional surveys in 2015-16 and 2018-19. *Lancet Public Health*. 2020; 5: e639-e49.
